# Characterisation of *Shigella* Spa33 and *Thermotoga* FliM/N reveals a new model for C‐ring assembly in T3SS

**DOI:** 10.1111/mmi.13267

**Published:** 2015-12-23

**Authors:** Melanie A. McDowell, Julien Marcoux, Gareth McVicker, Steven Johnson, Yu Hang Fong, Rebecca Stevens, Lesley A. H. Bowman, Matteo T. Degiacomi, Jun Yan, Adam Wise, Miriam E. Friede, Justin L. P. Benesch, Janet E. Deane, Christoph M. Tang, Carol V. Robinson, Susan M. Lea

**Affiliations:** ^1^ Present address: Biochemistry Centre (BZH)University of HeidelbergIm Neuenheimer Feld 328HeidelbergGermany; ^2^ Present address: Institute of Pharmacology and Structural Biology (IPBS)CNRSUMR 5089, 205 Route de NarbonneToulouseFrance; ^3^ Present address: Cambridge Institute for Medical ResearchWellcome Trust/MRC Bldg.Addenbrooke's HospitalHills RoadCambridgeUK; ^4^ Sir William Dunn School of PathologyUniversity of OxfordOxfordUK; ^5^ Department of ChemistryUniversity of OxfordOxfordUK

## Abstract

Flagellar type III secretion systems (T3SS) contain an essential cytoplasmic‐ring (C‐ring) largely composed of two proteins FliM and FliN, whereas an analogous substructure for the closely related non‐flagellar (NF) T3SS has not been observed *in situ*. We show that the *spa33* gene encoding the putative NF‐T3SS C‐ring component in *S* *higella flexneri* is alternatively translated to produce both full‐length (Spa33‐FL) and a short variant (Spa33‐C), with both required for secretion. They associate in a 1:2 complex (Spa33‐FL/C_2_) that further oligomerises into elongated arrays *in vitro*. The structure of Spa33‐C_2_ and identification of an unexpected intramolecular pseudodimer in Spa33‐FL reveal a molecular model for their higher order assembly within NF‐T3SS. Spa33‐FL and Spa33‐C are identified as functional counterparts of a FliM–FliN fusion and free FliN respectively. Furthermore, we show that *T* *hermotoga maritima* FliM and FliN form a 1:3 complex structurally equivalent to Spa33‐FL/C_2_, allowing us to propose a unified model for C‐ring assembly by NF‐T3SS and flagellar‐T3SS.

## Introduction

Non‐flagellar type III secretion systems (NF‐T3SS) are essential for initiating infection by many pathogenic Gram‐negative bacteria (Cornelis, 2006). These include several enteropathogenic species such as *Salmonella*, *Escherichia*, *Yersinia* and, the primary focus of this study, *Shigella*, which invades a variety of cells in the intestinal tract and causes over one million deaths annually from bacterial dysentery or shigellosis (Kotloff *et al*., 1999). This large protein export apparatus (Fig. S1) is composed of ∼ 25 different proteins with both species specific and unified Sct names and functions as a molecular syringe, injecting virulence factors from the bacterial cytoplasm directly into the host‐cell (Marlovits and Stebbins, 2010; Abrusci *et al*., 2014). Secretion by NF‐T3SS occurs by a defined hierarchy, with a secretion‐competent complex (comprising a dual membrane‐spanning basal body, cytoplasmic components and an export apparatus) enabling sequential assembly of a hollow extracellular needle, insertion of a contiguous pore into the eukaryotic cell membrane and transport of effectors through this conduit (Diepold and Wagner, 2014).

Early visualisations of *Shigella flexneri* membrane ghosts revealed the NF‐T3SS to have a prominent cytoplasmic bulb at the base of the basal body, likely composed of the soluble components that control and regulate secretion (Blocker *et al*., 1999). In particular, the protein Spa33 (SctQ), which is essential for secretion and localises to the base of the *S. flexneri* NF‐T3SS *in situ* via association with the basal body (Morita‐Ishihara *et al*., 2006; Barison *et al*., 2012), has been proposed to form a substructure termed the cytoplasmic‐ring (C‐ring) (Morita‐Ishihara *et al*., 2006). The *Salmonella* pathogenicity island (SPI)‐1 orthologue SpaO has the propensity to form large molecular weight complexes that interact with NF‐T3SS chaperone‐substrate complexes with differential affinities, leading to the suggestion that this C‐ring may act as a ‘sorting platform’ to establish the correct secretion hierarchy (Lara‐Tejero *et al*., 2011). In addition, interactions between SctQ and the other essential cytoplasmic components SctN, SctL and SctK (Fig. S1) were identified in various species (Jackson and Plano, 2000; Johnson and Blocker, 2008; Johnson *et al*., 2008; Biemans‐Oldehinkel *et al*., 2011; Lara‐Tejero *et al*., 2011) and shown to be required for their colocalisation at the base of the *Yersinia* NF‐T3SS (Diepold *et al*., 2010), indicating the C‐ring likely forms part of a significant cytoplasmic structure.

In the first cryoelectron tomography structures of the NF‐T3SS from *Shigella*, *Yersinia* and *Salmonella*, a C‐ring was notably absent, despite clear density for the SctN ATPase in the cytoplasm (Kawamoto *et al*., 2013; Kudryashev *et al*., 2013). However, in a more recent *in situ* structure of the NF‐T3SS from *S. flexneri* minicells, a more extensive cytoplasmic substructure was observed, whereby six SctL spokes radiate from the central SctN hub and connect to the C‐ring component (Hu *et al*., 2015). Although Hu *et al*. (2015) observe only discrete pods of density for Spa33 instead of a ‘C‐ring’, *in vivo* fluorescent measurements subsequently showed ∼ 22 copies of the *Yersinia* orthologue are present at the base of the NF‐T3SS and undergoing rapid exchange with a cytosolic pool (Diepold *et al*., 2015), indicating a more extensive yet unstable C‐ring substructure may still exist.

The NF‐T3SS shares a similar overall architecture and many individual components with the bacterial flagellum, which uses a T3SS to assemble an extracellular hook and filament (Büttner, 2012). In particular, the flagellar‐T3SS has a C‐ring that is comparatively better characterised, with a cryoelectron microscopy (EM) reconstruction of the entire substructure showing ∼ 34‐fold symmetry (Thomas *et al*., 2006). The C‐terminus of Spa33 shows weak sequence homology to the SpoA domains of FliM and FliN that form the flagellar C‐ring along with FliG. Recently, it has been shown for the orthologues *Yersinia* YscQ and *Salmonella* SPI‐2 SsaQ that an alternative translation initiation site exists within the gene, leading to the production of both the full‐length protein and a C‐terminal fragment (Yu *et al*., 2011; Bzymek *et al*., 2012). Together with the structural similarity between the homodimers formed by the YscQ C‐terminal variant (YscQ‐C) and FliN SpoA domains (Brown *et al*., 2005; Bzymek *et al*., 2012), it has been suggested that the full‐length and C‐terminal proteins are likely to be equivalent to FliM and FliN respectively and are therefore both integral components of the putative NF C‐ring as in the flagellar C‐ring. Indeed, both YscQ and YscQ‐C are required for assembly and function of the *Yersinia* NF‐T3SS (Bzymek *et al*., 2012; Diepold *et al*., 2015), although a chaperone role for the shorter variant has also been proposed (Yu *et al*., 2011).

Although initially identified as part of the rotational switch complex, the flagellar C‐ring is also essential for protein secretion (Vogler *et al*., 1991) and is involved in interactions with the SctL homologue FliH (Paul and Blair, 2006; Minamino *et al*., 2009) and indirectly with the flagellar basal body (Thomas *et al*., 2006), suggesting similarities to the role of the C‐ring within the NF‐T3SS. However, the contiguous C‐ring robustly associated with the flagellar‐T3SS (Thomas *et al*., 2006) is clearly different from the recently observed pod‐like structures formed by Spa33 at the base of the *Shigella* NF‐T3SS (Hu *et al*., 2015). Furthermore, full‐length YscQ has been shown to interact with dimeric YscQ‐C to form a 1:2 complex (Bzymek *et al*., 2012), which is at odds with the proposed 1:4 stoichiometry for FliM and FliN (Brown *et al*., 2005). Together with the additional role of the flagellar C‐ring in torque generation and direction switching during flagellum rotation, it is possible that the C‐ring proteins of the NF‐T3SS could adopt a different arrangement to fulfil different functional requirements. Also, *S. flexneri spa33* has neither a start codon nor an unambiguous ribosome binding site (RBS) in the vicinity of the alternative translation initiation site found in orthologous genes, leading to suggestions that this mechanism for producing a FliN‐like C‐terminal variant may not be conserved across all NF‐T3SS (Bzymek *et al*., 2012). Therefore, many aspects of the structure and function of the *S. flexneri* C‐ring could not be assumed or predicted based on our current knowledge of orthologues from NF‐ and flagellar‐T3SS, requiring further characterisation to determine the extent to which C‐ring structure has diversified between systems.

In this study, we show that alternative translation occurs to generate full‐length Spa33 (Spa33‐FL) and a shorter C‐terminal fragment (Spa33‐C) within *S. flexneri*, with the production of both being required for assembly and function of the NF‐T3SS. High resolution biophysical studies demonstrate that Spa33‐FL and Spa33‐C associate to form a minimal 1:2 complex that then undergoes further oligomerisation to produce elongated arrays. We also identify an additional SpoA domain in the SctQ sequences, allowing us to propose a new molecular model for C‐ring assembly. Using native mass spectrometry (MS), we reanalyse the assembly of the canonical flagellar C‐ring of *Thermotoga maritima* and demonstrate that its sub‐complexes are built from a 1:3 ratio of FliM/FliN that is consistent with our Spa33‐FL/C_2_ model, thereby supporting a single, unified model for assembly of the NF and flagellar C‐ring.

## Results

### Spa33‐FL and Spa33‐C are alternative translation products of the same gene and are both required to form a functional NF‐T3SS

**Figure 1:**
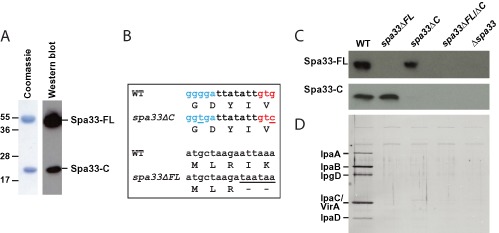
Spa33‐C is an alternative expression product of *S* *. flexneri* spa33 and forms a complex with Spa33‐FL that is required for T3SS function *in vivo*. A. Equivalent sections from a Coomassie stained gel and α‐Spa33 Western blot showing bands for the purified complex of Spa33‐FL and Spa33‐C. Samples were separated on a 15% SDS‐PA gel and α‐Spa33 was used at 1:50 000 dilution in blocking buffer. B. Nucleotide and protein sequences for selected regions of the *spa33* gene from WT *S* *. flexneri*. The RBS (blue) and start codon (red) at the predicted alternative translation initiation site for Spa33‐C are shown within the nucleotide sequence. The mutations in the *spa33* sequences of *spa33Δ* *C* and *spa33Δ* *FL* strains and their affect on the amino acid sequence are shown relative to the WT sequence. C. α‐Spa33 Western blot of whole cell lysate taken from WT, *spa33Δ* *FL*, *spa33Δ* *C*, *spa33Δ* *FL* */Δ* *C* and *Δspa33* strains of *S* *. flexneri* showing *in vivo* levels of Spa33‐FL and Spa33‐C. Samples were separated on a 15% SDS‐PA gel and α‐Spa33 was used at 1:1000 dilution in blocking buffer. D. CR induction assay for WT, *spa33Δ* *FL*, *spa33Δ* *C*, *spa33Δ* *FL* */Δ* *C* and *Δspa33* strains of *S* *. flexneri*. Cell cultures were supplemented with 0.2 mg ml^−1^ CR, and samples taken from the supernatant were separated on a 10% SDS‐PA gel and silver‐stained. The position of early effectors within the characteristic gel profile is shown.

Spa33 with an N‐terminal His‐tag was expressed recombinantly in *Escherichia coli*, allowing purification via Ni‐affinity and size exclusion chromatography (SEC). Two proteins were observed to co‐purify throughout (Fig. 1A), with masses of 35.6 kDa and 11.6 kDa defined by MS. Western blotting of the purified complex with anti‐Spa33 polyclonal antibodies raised against residues 208–293 of the protein (α‐Spa33) showed both species to contain this C‐terminal region of Spa33 (Fig. 1A). The larger species was therefore able to be assigned as tagged full‐length Spa33 (Spa33‐FL), whereas the size of the smaller species was consistent with residues 192–293 at the extreme C‐terminus of Spa33, hereafter referred to as Spa33‐C. In addition, both Spa33‐FL and Spa33‐C were detected by α‐Spa33 within wild‐type *S. flexneri* (WT; Table 1), indicating Spa33‐C is a physiologically relevant species (Fig. 1C).

**Table 1:** *S* *. flexneri* strains used in this study

Strain	Description	Source
WT	Wild‐type M90T, serotype 5a	Sansonetti *et al*. (1982)
*spa33::sacB‐kanR*	GMCT113, Kan^r^	This study
*spa33ΔFL*	GMCT129 (allelic exchange of GMCT113 with pGM133)	This study
*spa33ΔC*	GMCT131 (allelic exchange of GMCT113 with pGM134)	This study
*spa33ΔFL/C*	GMCT133 (allelic exchange of GMCT113 with pGM136)	This study
*Δspa33*	GMCT135 (allelic exchange of GMCT113 with pGM135)	This study

We reasoned that Spa33‐C could either be an alternative product of the *spa33* gene or produced by post‐translational cleavage of Spa33‐FL. The experimentally determined mass of Spa33‐C (11630 ± 9 Da; data not shown) indicated this species would be expected to have a formylmethionine residue at the N‐terminus (11634 Da) rather than the valine residue encoded by the GTG codon at this position (11574 Da). Further inspection of the *spa33* gene sequence revealed a purine‐rich putative RBS in the expected position upstream of the first codon of Spa33‐C (Fig. 1B, Fig. S2), suggesting Spa33‐C is produced from a distinct translation start site to Spa33‐FL. To test this hypothesis, allelic exchange was used to introduce silent mutations in both the putative RBS and alternative start codon (Fig. 1B) within the *spa33* gene of *S. flexneri*, generating strain *spa33ΔC* (Table 1). A greatly diminished level of Spa33‐C was detected by α‐Spa33 within *spa33ΔC* relative to the WT, whereas the level of Spa33‐FL remained the same (Fig. 1C). Conversely, a *spa33ΔFL* strain (Table 1), which has tandem stop codons inserted after the first three codons of the *spa33* gene (Fig. 1B), was able to produce Spa33‐C in the absence of Spa33‐FL (Fig. 1C). Together these data show that Spa33‐C is translated independently of Spa33‐FL via the identified alternative initiation site within the *spa33* gene.

The basal functionality of the *S. flexneri* T3SS can be assessed via addition of Congo red (CR) to the growth medium, which mimics host‐cell contact and induces secretion of early effectors by the WT strain (Fig. 1D) (Bahrani *et al*., 1997). Neither the *spa33ΔC* nor the *spa33ΔFL* strain displayed secretion upon CR induction (Fig. 1D), phenotypically mimicking a *Δspa33* strain (Table 1) and indicating that production of both Spa33‐FL and Spa33‐C are required for assembly of a functional T3SS.

### Structural characterisation of Spa33‐C

**Figure 2:**
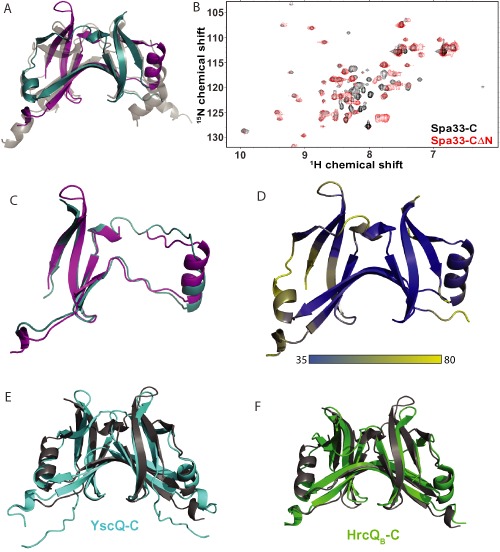
Spa33‐C forms a highly intertwined homodimer with unstructured N‐terminal extensions. A. The crystal structure of Spa33_208‐293_ shown as a cartoon representation. Chain A and B are coloured teal and purple respectively. The Spa33_208‐293_ dimer is superimposed with the *T* *. maritima* FliN structure (pdb id 1YAB), shown in grey. B. Overlay of ^1^ H,^15^ N‐HSQC spectra for 100 μM Spa33‐C (black) and Spa33‐CΔN (red) collected under the same buffer conditions. C. Superimposition of chain A (teal) and chain B (purple) of the Spa33_208‐293_ dimer (1.70 Å RMSD, 64 C ^α^ atoms). D. The structure of Spa33_208‐293_ coloured according to the C ^α^ B‐factor (Å^2^). Superimposition of the Spa33_208‐293_ dimer (grey) with the structures of E *Y* *. pseudotuberculosis* YscQ‐C (turquoise; pdb id 3UEP) and F *P* *. syringae* HrcQ_B_‐C (green; pdb id 1O9Y).

The C‐terminal 86 residues of Spa33 (Spa33_208‐293_) were expressed with a C‐terminal His‐tag in *E. coli*, purified to homogeneity and the tag removed for successful crystallisation trials. The 2.3 Å resolution crystal structure of Spa33_208‐293_ was subsequently determined by molecular replacement (Table 2). The structure shows a SpoA domain that forms the expected saddle‐shaped homodimer with an approximate twofold rotational symmetry axis (Fig. 2A). Regions of notable asymmetry (Fig. 2C) have high B‐factors (Fig. 2D), suggesting conformational flexibility accounts for major differences between the dimer halves. Each copy of Spa33_208‐293_ is highly intertwined within the dimer, with the fold being highly homologous to that observed in the crystal structures of orthologous proteins. Indeed superimposition of Spa33_208‐293_ with *T. maritima* FliN (Brown *et al*., 2005) (2.18 Å RMSD, 126 Cα atoms)(Fig. 2A), *Yersinia pseudotuberculosis* YscQ‐C (Bzymek *et al*., 2012) (2.09 Å RMSD, 135 Cα atoms) (Fig. 2E) and *Pseudomonas syringae* HrcQ_B_‐C (Fadouloglou *et al*., 2004) (2.40 Å RMSD, 133 Cα atoms) (Fig. 2F) shows a remarkably similar arrangement of secondary structure elements.

**Table 2:** Data collection and refinement statistics

Data collection	
λ (Å)	0.9795
Space group	*P*2_1_
Unit cell dimensions (Å, °)	51.3, 27.8, 104.4, 90, 90.03, 90
Resolution (Å)^a^	51.3–2.3 (2.38–2.30)
Number of unique reflections	13523 (1286)
Multiplicity^a^	3.6 (3.6)
Completeness (%)^a^	99.5 (99.1)
R_merge_ a	0.042 (0.77)
R_pim_ a	0.029 (0.55)
CC1/2	0.999 (0.660)
Average I/σ(I)^a^	21.3 (2.1)
*Refinement*	
R_work_/R_free_	17.4/21.0 (32.4/36.5)
RMSDs	
Bond lengths (Å)	0.007
Bond angles (°)	1.03
No. of atoms	
Protein	2273
Ligand	16
Water	65
Ramachandran^b^	
Favoured (%)	93.4
Allowed (%)	100
Disallowed (%)	0
Average B‐factor	56.9
Molprobity score	2.65

a Values in brackets are for the highest resolution shell.

b Determined using MolProbity (Davis *et al*., 2007).

The structure of Spa33_208‐293_ may provide a good model for the physiologically relevant species Spa33‐C, which has only an additional 28 residues at the N‐terminus not visible in the crystal structure. To test this, full‐length Spa33‐C and a truncated construct comprising only those residues visible in the crystal structure (Spa33‐CΔN) were purified as ^15^N‐labelled proteins for nuclear magnetic resonance (NMR) spectroscopy. An overlay of the ^1^H,^15^N‐HSQC spectra obtained for each protein shows a high number of coincident peaks (Fig. 2B), which have a good chemical shift dispersion in both dimensions and provide further evidence that this shared region likely has the highly structured fold determined by X‐ray crystallography. In contrast, the additional peaks present for Spa33‐C that correspond to residues within the uncharacterised N‐terminal extension are collapsed into the central region of the spectrum (Fig. 2B). This suggests that the N‐terminal 28 residues of Spa33‐C are flexible and unstructured, further evidenced by their absence in the crystal structures of Spa33_208‐293_ and other orthologous proteins (Fadouloglou *et al*., 2004; Brown *et al*., 2005; Bzymek *et al*., 2012).

### Spa33‐FL and Spa33‐C associate to form a 1:2 complex that undergoes further oligomerisation *in vitro*

**Figure 3:**
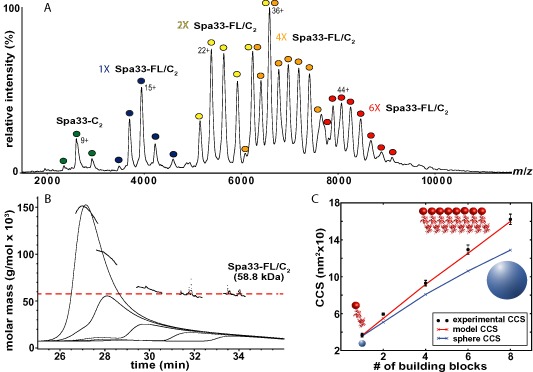
Spa33‐FL and Spa33‐C interact to form a 1:2 complex that undergoes further oligomerisation into elongated arrays *in vitro*. A. Mass spectrum of His‐Spa33‐FL/Spa33‐C complexes obtained under non‐denaturing conditions showing the presence of Spa33‐C_2_ (dark green) and Spa33‐FL/C_2_ (dark blue) building blocks. The other species correspond to even assemblies of the Spa33‐FL/C_2_ subcomplex, with dimers (yellow), tetramers (orange) and hexamers (red) distinguishable. For each assigned complex, the charge state of the most intense peak is shown and a comparison of experimental and theoretical molecular mass is given in Table 3. B. Analysis of the oligomeric state of His‐Spa33‐FL/Spa33‐C complexes by SEC and in‐line MALS. The left axis represents the molecular mass at any given point of the chromatogram. Elution profiles are shown for the main elution peak for the dilution series (from left to right) 60 μM, 30 μM, 15 μM, 7.5 μM and 3.7 μM Spa33‐FL/C_2_ and the expected molar mass for Spa33‐FL/C_2_ is marked. C. Comparison of experimental CCS (black) measured for each complex visible in the native mass spectrum in A with theoretical values for spherical (blue) and linear (red) assemblies of Spa33‐FL/C_2_. To obtain theoretical CCS values for spherical aggregates of Spa33‐FL/C_2_, the diameter of a sphere having the same CCS as the experimental monomer was determined and then used to calculate the CCS of spheres with 2–8 times bigger volume. In order to obtain theoretical CCS values for linear assemblies of Spa33‐FL/C_2_, CSS was directly calculated from the spiral arrangement of SpoA dimers observed in the Spa33_208‐293_ crystal lattice (red cartoon representation equivalent to the molecular model in Fig. 5D) and incremented to account for the missing mass of the Spa33‐FL N‐terminal domain (red sphere).

To ascertain the stoichiometry and nature of the interaction between Spa33‐FL and Spa33‐C, the purified complex was subjected to native MS. All detectable Spa33‐FL was found to be associated with Spa33‐C, with its smallest complex being accounted for by one copy of Spa33‐FL and two copies of Spa33‐C (Spa33‐FL/C_2_) (Fig. 3A). As further verification, SEC‐MALS was carried out with various concentrations of the purified complex, showing the smallest observed species to have a molar mass consistent with Spa33‐FL/C_2_ (Fig. 3B). Therefore, a 1:2 complex is probably the minimal unit formed by Spa33‐FL and Spa33‐C, in agreement with findings for the *Yersinia* C‐ring proteins (Bzymek *et al*., 2012).

Both the native mass spectrum (Fig. 3A) and SEC‐MALS trace (Fig. 3B) for purified Spa33‐FL/C_2_ clearly show that this complex is able to oligomerise further, with high molecular weight species comprising up to six copies of Spa33‐FL/C_2_ able to be unambiguously assigned in the native mass spectrum (Fig. 3A). In order to probe the shape of these large oligomers of Spa33‐FL/C_2_, native MS was coupled with ion mobility (IM‐MS), allowing species to be separated on the basis of their collisional cross‐section (CCS). The experimentally determined CCS values for oligomers of Spa33‐FL/C_2_ were seen to deviate from theoretical CCS values for spherical aggregates of the complex (Fig. 3C). Furthermore, SEC‐MALS suggested that concentration‐dependent Spa33‐FL/C_2_ oligomerisation was highly dynamic and reversible (Fig. 3B), suggesting that Spa33‐FL/C_2_ is unlikely to be undergoing uncontrolled aggregation in solution. Therefore, the data suggest that Spa33‐FL/C_2_ acts as a building block in the formation of large, ordered oligomers, as would be required to form a structure akin to the flagellar C‐ring.

### The flagellar C‐ring proteins FliM and FliN form a 1:3 complex in vitro

As *S. flexneri* Spa33‐FL/Spa33‐C appear to mimic characteristics of FliM/FliN respectively from the flagellar‐C‐ring, we considered whether the complexes formed by these proteins would maintain similar interactions and oligomeric states. However, our finding that the minimal unit formed by Spa33‐FL and Spa33‐C is a 1:2 complex differs from the current model for flagellar C‐ring assembly, where analytical ultracentrifugation (AUC) and cross‐link based modelling suggest a 1:4 association of FliM and FliN (Brown *et al*., 2005). Therefore, we sought to re‐evaluate the stoichiometry of FliM/FliN complexes *in vitro* using native MS, as this technique is able to distinguish between different complexes within a mixture and yields much smaller experimental errors on molar mass measurements (Marcoux and Robinson, 2013).

**Figure 4:**
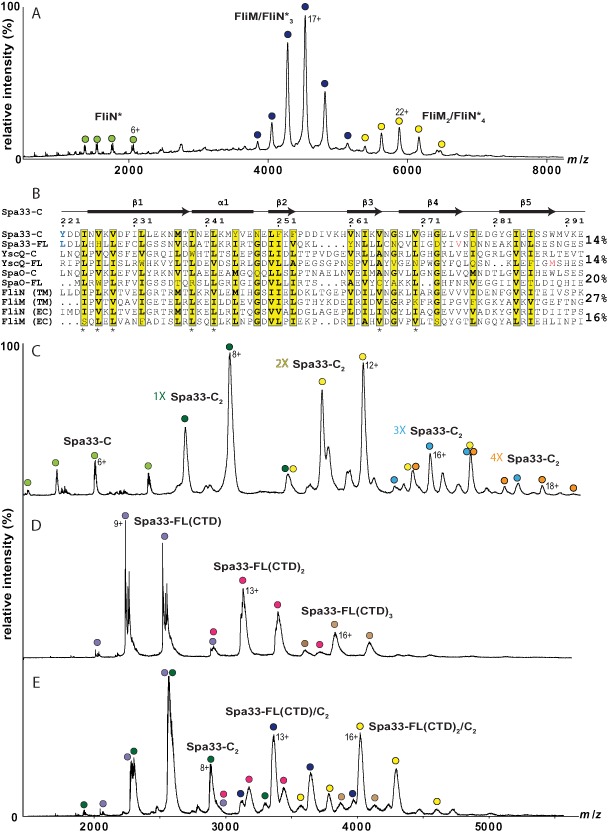
*T. maritima* FliM and FliN* interact to form a 1:3 complex that is analogous to the 1:2 complex formed by Spa33‐FL(CTD) and Spa33‐C. A. Mass spectrum of His‐FliM/FliN* complexes obtained under non‐denaturing conditions showing the presence of FliN* (light green), FliM/FliN*_3_ (dark blue) and FliM_2_/FliN*_4_ building blocks (yellow). The peak splitting for the FliN* charge state is due to the partial loss of the N‐terminal methionine residue. For each assigned complex, the charge state of the most intense peak is shown and a comparison of experimental and theoretical molecular mass is given in Table 3. B. Multiple sequence alignment of the SpoA2 domain of Spa33‐C and the SpoA1 domain of Spa33‐FL with orthologues from the NF‐T3SS in *Y* *. pseudotuberculosis* (YscQ) and *S* *. typhimurium* SPI‐1 (SpaO) and with FliN/FliM from the flagellar‐T3SS in *T* *. maritima* (TM) and *E* *. coli* (EC), performed using ClustalW2 (Chenna *et al*., 2003) and represented using ESPript 3.0 (http://espript.ibcp.fr) (Gouet *et al*., 2003). Conserved similar residues are shown in yellow, with asterisks denoting those residues within the conserved dimer–dimer interface and the key contact residues between Spa33‐C and Spa33‐FL that were mutated in this study, Tyr_221_ and Leu_141_, being shown in blue. The secondary structure and residue numbering for Spa33‐C are shown above the sequence and alternative translation start sites within Spa33‐FL and YscQ‐FL are shown in red. The pairwise sequence identity between the SpoA2 and SpoA1 domain is given beside each pair of proteins from either the NF‐ or flagellar‐T3SS. C. Mass spectrum obtained for Spa33‐C under non‐denaturing conditions. Multimeric assemblies are made of dimeric subunits, with monomers (dark green), dimers (yellow), trimers (blue) and tetramers (orange) distinguishable. Monomeric Spa33‐C (light green) is present due to gas phase dissociation. For each assigned complex, the charge state of the most intense peak is shown and a comparison of experimental and theoretical molecular mass is given in Table 3. D. Mass spectrum of Spa33‐FL(CTD) complexes obtained under non‐denaturing conditions showing the presence of monomer (purple), dimer (magenta) and trimer (brown). For each assigned complex, the charge state of the most intense peak is shown and a comparison of experimental and theoretical molecular mass is given in Table 3. E. Mass spectrum of Spa33‐FL(CTD)/C complexes obtained under non‐denaturing conditions. In addition to the oligomers of isolated Spa33‐FL(CTD) observed in B, Spa33‐C_2_ (dark green), Spa33‐FL(CTD)/C_2_ (blue) and Spa33‐FL(CTD)_2_/C_2_ (yellow) species are also present. For each assigned complex, the charge state of the most intense peak is shown and a comparison of experimental and theoretical molecular mass is given in Table 3.

The 1:4 model for FliM and FliN association was originally proposed based on data acquired from the *T. maritima* C‐ring proteins (Brown *et al*., 2005). Subsequently, this *T. maritima* ‘FliN’ construct has been characterised as the C‐terminal 132 residues of a FliY protein, an additional component of the flagellar C‐ring that replaces or acts in conjunction with a FliN‐like protein in a subset of bacteria (Sircar *et al*., 2013). Using native MS, we found that *T. maritima* FliM/FliN (Fig. S3B) and FliM/FliY (Fig. S3C) existed as a complicated mixture of complexes, largely due to the partial incorporation of a smaller species corresponding to residues 235–343 of FliY (FliN*) (Fig. S3A). Intriguingly, the experimentally determined molecular mass of FliN* (12314 ± 1 Da) (Table 3) suggested the N‐terminal residue was likely to be formylmethionine (12302 Da) instead of valine (12242 Da) and a 30 nucleotide stretch of purines lies upstream of the start site for this species. Therefore, FliN* seems likely to be a product of alternative translation initiation of the *fliY* gene (Fig. S2), hinting that *T. maritima* could also synthesise a FliN‐like protein in a similar manner to the production of Spa33‐C by *S. flexneri*. When only FliM/FliN* were coexpressed and purified it was found to predominantly form 1:3 complexes by native MS (Fig. 4A), which was at odds with both our observation of a 1:2 complex for Spa33‐FL/Spa33‐C and the previously characterised *T. maritima* FliM/FliN 1:4 complex (Brown *et al*., 2005).

**Table 3:** Molecular masses determined by native mass spectrometry in this study

Complex	Experimental molecular mass (Da)	Theoretical molecular mass (Da)
*Spa33‐FL/C_2_ spectrum* a		
Spa33‐C_2_	23180 ± 73	23212
Spa33‐FL/C_2_	58992 ± 89	58757
(Spa33‐FL/C_2_)_2_	117786 ± 34	117514
(Spa33‐FL/C_2_)_4_	235645 ± 78	235028
(Spa33‐FL/C_2_)_6_	353804 ± 288	352542
*T. maritima FliM/FliN spectrum* b		
FliN*	12313 ± 1	12302
FliN	15144 ± 1	15146
FliN_2_	30295 ± 14	30292
FliM/FliN_2_/FliN*	82609 ± 13	82645
FliM/FliN_3_	85446 ± 4	85489
FliM_2_/FliN_2_/FliN*_2_	133660 ± 24	134998
FliM_2_/FliN_3_/FliN*	137724 ± 49	137842
FliM_2_/FliN_4_	140504 ± 20	140686
FliM_3_/FliN_4_/FliN*_2_	205128 ± 271	205341
*T. maritima FliM/FliY spectrum* b		
FliM/FliY	77830 ± 7	78048
FliM/FliY/FliN*_2_	102458 ± 9	102653
FliM/FliY/FliN/FliN*	105299 ± 23	105496
FliM/FliY_2_/FliN*	128038 ± 14	128347
FliM/FliY_2_/FliN/FliN*_2_	154760 ± 45	155795
FliM/FliY_2_/FliN_2_/FliN*	157606 ± 26	158639
FliM_2_/FliY_2_/FliN*_2_	180416 ± 43	180700
FliM_2_/FliY_2_/FliN/FliN*	183256 ± 41	183544
FliM_2_/FliY_3_/FliN*	206064 ± 40	206395
T. maritima FliM/FliN* spectrum b		
FliN*	12284 ± 1 and 12415 ± 1	12282 and 12413 (± N‐terminal Met)
FliM/FliN*_3_	77075 ± 32	76897 and 77293
FliM_2_/FliN*_4_	129488 ± 16	129230 and 129754
*Spa33‐C spectrum* c, d		
Spa33‐C	12023 ± 1	12021
Spa33‐C_2_	24134 ± 3	24042
(Spa33‐C_2_)_2_	48265 ± 7	48084
(Spa33‐C_2_)_3_	72390 ± 15	72126
(Spa33‐C_2_)_4_	96552 ± 47	96168
*Spa33‐FL(CTD)/Spa33‐C spectrum* a		
Spa33‐FL(CTD)	20467 ± 7	20449
Spa33‐FL(CTD)_2_	40941 ± 7	40898
Spa33‐FL(CTD)_3_	61669 ± 85	61347
Spa33‐C_2_	23005 ± 15	22984
Spa33‐FL(CTD)/C_2_	43465 ± 4	43433
Spa33‐FL(CTD)_2_/C_2_	63976 ± 20	63882
*Spa33‐FL/C_2_(Y221R) spectrum* a		
Spa33‐FL/C_2_(Y221R)	59106 ± 19	58772
(Spa33‐FL/C_2_(Y221R))_2_	118780 ± 19	117544
*Spa33‐FL/Cstrep_2_ spectrum* a		
Spa33‐Cstrep_2_	25217 ± 7	25064
Spa33‐FL/Cstrep_2_	60819 ± 54	60645
(Spa33‐FL/Cstrep_2_)_2_	121627 ± 69	121290
(Spa33‐FL/Cstrep_2_)_4_	243761 ± 159	242580
*Spa33‐FL/Ctrx_2_ spectrum* a		
Spa33‐Ctrx_2_	47465 ± 6	46991
Spa33‐FL/Ctrx_2_	83143 ± 1	82600
(Spa33‐FL/Ctrx_2_)_2_	166123 ± 1	165200
*Spa33‐FL/Cmbp_2_ spectrum* a		
Spa33‐Cmbp	51737 ± 3	51687
Spa33‐Cmbp_2_	103467 ± 2	103374
Spa33‐FL/Cmbp_2_	138988 ± 4	138955

a Native protein sequence of Spa33‐FL or Spa33‐FL(CTD) construct has additional N‐terminal MGSSHHHHHHSSGLVPRGSH affinity tag.

b Native protein sequence of FliM construct has additional N‐terminal MGSSHHHHHHSSGLVPRGSH affinity tag.

c Spectrum acquired with ^15^N‐labelled protein; theoretical molecular mass calculated assuming 100% labelling efficiency.

d Native protein sequence of Spa33‐C construct has additional GSH N‐terminal extension remaining after thrombin cleavage of affinity tag.

### Spa33‐FL and orthologous NF‐T3SS proteins correspond to a fusion of FliM and FliN

**Figure 5:**
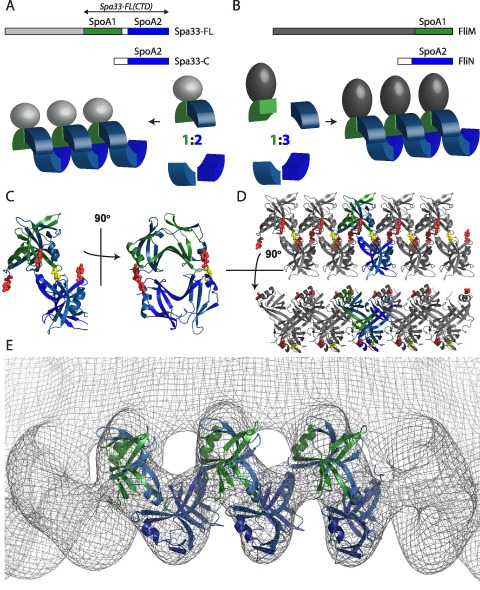
A unified model for C‐ring assembly in the NF‐ and flagellar‐T3SS. A. Model for C‐ring assembly by Spa33‐FL/C_2_. SpoA1, SpoA2 and the structurally uncharacterised N‐terminal domain of Spa33‐FL are coloured with respect to the primary sequence and tertiary structure schematic in green, blue and grey, respectively, whereas SpoA2 of Spa33‐C is coloured blue. An intramolecular pseudodimer formed by Spa33‐FL SpoA1 and SpoA2 interacts with an intermolecular homodimer of Spa33‐C SpoA2 to form a 1:2 complex that can subsequently oligomerise to form a spiral arrangement of dimers. The portion of Spa33‐FL found within the Spa33‐FL(CTD) construct is indicated. B. Model for C‐ring assembly by FliM/FliN_3_. SpoA1 and the structurally characterised N‐terminal domain of FliM are coloured with respect to the primary sequence and tertiary structure schematic in green and grey, respectively, whereas SpoA2 of FliN is coloured blue. An intermolecular heterodimer formed by FliM SpoA1 and FliN SpoA2 interacts with an intermolecular homodimer of FliN SpoA2 to form a 1:3 complex that can subsequently oligomerise to form a spiral arrangement of dimers. C. Molecular model for the Spa33‐FL/C_2_ complex. A model for the intramolecular pseudodimer formed by SpoA1 and SpoA2 of Spa33‐FL was made by replacing chain A of Spa33_208‐293_ with a SCWRL homology model (Canutescu *et al*., 2003) for Spa33‐FL SpoA1, constructed using the sequence alignment shown in Fig. 4B. The model for the 1:2 complex was made by preserving crystal contacts observed between Spa33_208‐293_ dimers and replacing one copy of Spa33_208‐293_ with the model for the Spa33‐FL intramolecular dimer. Sites mutated to probe assembly are highlighted as red spheres (Tyr_221_ – two copies from Spa33‐C chains and one from the SpoA2 domain of Spa33‐FL) or yellow spheres (Leu_141_ – in the SpoA1 domain of Spa33‐FL, at the position equivalent to Tyr_221_). Formation of the interface within the 1:2 assembly is built from Tyr_221_ within a Spa33‐C chain and Leu_141_ within the SpoA1 domain in Spa33‐FL. D. Molecular model for linear arrays of Spa33‐FL/C_2_. A lateral arrangement of the model shown in C was produced based on contacts observed in the Spa33_208‐293_ crystal. Assembly of the 1:2 complex into the linear array is driven by an interface where two Tyr_221_ residues meet (one from Spa33‐C and one from the SpoA2 domain of Spa33‐FL). E. The Spa33‐FL/C_2_ model shows a good correlation with the cryo‐EM map of the flagellar C‐ring. Molecular models for the Spa33‐FL/C_2_ complex shown in C was manually positioned in the density corresponding to the cytoplasmic edge of the C‐ring in the C34 *S* *. typhimurium* EM map contoured to 1σ (Thomas *et al*., 2006) (EMDB 1887).

Given the sequence and structural homology between the SpoA domains of Spa33‐C and FliN*, our finding that Spa33‐FL and Spa33‐C associate with a different stoichiometry from FliM and FliN* led us to suspect a fundamental difference in the structures formed by Spa33‐FL and FliM. We therefore searched the PDB using the fold and function assignment server (Jaroszewski *et al*., 2005) with the sequences of Spa33‐FL and homologues from other NF‐T3SS. Searching with residues 1–217 of *Y. pseudotuberculosis* YscQ‐FL returned the most significant alignments between an approximately 60 amino acid string immediately N‐terminal to the well‐characterised SpoA domain and the SpoA domains of HrcQ_B_‐C (score ‐13.0), YscQ‐C (score ‐12.1) and FliN (score −10.3). Multiple sequence alignment of the equivalent regions of Spa33‐FL, YscQ‐FL and *Salmonella typhimurium* SpaO‐FL with their respective C‐terminal SpoA domains showed a pairwise sequence identity of 14–20%, comparable with the similarity between the C‐terminal SpoA domains of flagellar FliM and FliN (Fig. 4B). Furthermore, this alignment revealed a striking conservation of similar residues coincident with the secondary structure elements of Spa33_208‐293_ (Fig. 4B), suggesting that Spa33‐FL and orthologous NF‐T3SS proteins have two SpoA domains arranged in tandem (Fig. 5A): the characterised C‐terminal SpoA domain that is also present in Spa33‐C (SpoA2), and an additional SpoA domain immediately upstream (SpoA1).

We therefore hypothesised that SpoA1 and SpoA2 within Spa33‐FL could form an intramolecular pseudodimer that is structurally homologous to Spa33‐C_2_, with the 14 residue linker of an appropriate length to bridge ∼ 37 Å between the N‐ and C‐termini of the dimer chains. Substitution of chain A of the Spa33_208‐293_ crystal structure with a SCWRL homology model (Canutescu *et al*., 2003) of Spa33‐FL SpoA1 creates a model for this predicted pseudodimer with the appropriate surface distribution of polar residues and a hydrophobic core comprising the majority of conserved residues (Fig. 4B). In order to test this model, we carried out a series of native MS experiments. First, as expected, untagged Spa33‐C was shown to be predominantly dimeric, with the larger oligomers also observed always being built from the Spa33‐C_2_ building block (Fig. 4C) (a small amount of monomeric Spa33‐C was observed, but the charge state distribution indicated it was formed by gas phase dissociation and did not exist in solution). On the other hand, a construct comprising only SpoA1 and SpoA2 of Spa33‐FL (Spa33‐FL(CTD); Fig. 5A) was found to be predominantly monomeric by native MS (Fig. 4D), indicating the 60 residues upstream of the alternative translation start site that encompass SpoA1 are sufficient to prevent intermolecular dimer formation by the Spa33 SpoA2 domain. Furthermore, Spa33‐FL(CTD) also showed the propensity to oligomerise in a similar manner to Spa33‐C_2_ (Fig. 4D), implying shared structural properties between the domains. Crucially, Spa33‐FL(CTD) and Spa33‐C were subsequently found to form a minimal 1:2 complex (Fig. 4E), suggesting that the 1:2 stoichiometry for Spa33‐FL and Spa33‐C association likely arises through the interaction of a Spa33‐C_2_ homodimer with the intramolecular pseudodimer of Spa33‐FL.

In contrast, flagellar FliM only has the single C‐terminal SpoA domain, with the sequence immediately upstream forming a CheC/CheX phosphatase fold (Park *et al*., 2006) (Fig. 5B). Therefore, this orphan SpoA domain of FliM would only be able to dimerise through intermolecular interactions. Given our finding that *T. maritima* FliM and FliN* form a 1:3 complex, we propose that FliM forms a heterodimer with FliN* that subsequently interacts with a homodimer of FliN*. Indeed, a FliM/FliY heterodimer was a prominent species observed in the native mass spectrum of FliM/FliY complexes (Fig. S3C). Therefore, the 1:2 complex of Spa33‐FL/Spa33‐C and 1:3 complex of FliM/FliN* are both likely to represent a dimer of dimers arrangement of SpoA domains and are therefore entirely consistent with a conserved building block in NF‐ and flagellar‐C‐ring assembly.

### A unified model for C‐ring assembly

In order to gain insights into the interaction of Spa33‐FL with Spa33‐C_2_, the intermolecular contacts of the Spa33_208‐293_ crystal were investigated and revealed a dimer–dimer interface formed by an equivalent surface of each homodimer, comprising regions proximal to the N‐terminus, C‐terminus and β3‐β4 turn from one chain and α1 from the other. This interaction buries a surface area of 508 Å^2^ and is stabilised by three hydrogen bonds and hydrophobic interactions between apolar residues that are conserved with SpoA1 of Spa33‐FL and orthologous domains (Fig. 4B). In particular, Tyr_221_ makes a significant contribution to the interaction, fitting into a hydrophobic pocket formed by the interacting dimer (Val_265_/Val_270_/Trp_289_/Val_291_ and Ile_239_/Leu_242_/Lys_243_ of opposing chains) (Fig. S4). Strikingly, this packing arrangement is also conserved in the HrcQ_B_‐C crystal lattice (Fadouloglou *et al*., 2004), despite the proteins only sharing 16% sequence identity, indicating a conserved mode of interaction by Spa33‐C orthologues (Fig. S5A) [this dimer–dimer interface is not present in the YscQ‐C crystal as the non‐native C‐terminal tag residues mask this interaction site (Fig. S5B) (Bzymek *et al*., 2012)]. Notably however, the conserved dimer–dimer interface is also similar to that proposed to be involved in formation of the ring‐shaped FliN tetramer (Paul and Blair, 2006) (Fig. S5C). In contrast to the closed tetramer of FliN, the Spa33_208‐293_ and HrcQ_B_‐C assemblies form an open lock washer structure, with the concave surfaces of the saddle‐shaped dimers being offset laterally by ∼ 55° with respect to each other.

By replacing one dimer with our model for the intramolecular pseudodimer formed by Spa33‐FL, this arrangement of Spa33_208‐293_ dimers allows a molecular model for the 1:2 complex of Spa33‐FL/Spa33‐C to be constructed (Fig. 5C). Crucially, this open arrangement of the Spa33‐FL/Spa33‐C_2_ complex would allow each dimer to undergo further dimer–dimer interactions via the equivalent interfaces. Indeed, a continuous spiral of Spa33_208‐293_ dimers is observed in the crystal lattice, providing a molecular model for formation of higher molecular weight oligomers by the analogous dimers of Spa33‐FL/C_2_ (Fig. 5A and D). Furthermore, theoretical CCS values calculated from this linear model showed excellent agreement with the experimental CCS values obtained for Spa33‐FL/C_2_ oligomers (Fig. 3C), providing evidence for the formation of such elongated arrays in the gas phase.

In this model for high molecular weight oligomers of Spa33‐FL/C_2_, the interaction surfaces provided by Spa33‐C_2_ are essentially equivalent, whereas the two provided by the Spa33‐FL intramolecular pseudodimer would vary depending on which regions of SpoA1 and SpoA2 combine to form the binding site. Therefore, the interaction between Spa33‐FL and Spa33‐C_2_ is likely to occur with two different affinities depending on which Spa33‐FL binding site is involved, with the stronger likely mediating formation of the stable 1:2 complex and the weaker promoting further oligomerisation of this building block. To probe these interfaces, we designed point mutations based on the crystal contacts and a homology model for the SpoA1 domain of Spa33‐FL. This model (Fig. 5C and D) revealed that there was a potential difference in the key contacting residue making up these two interfaces with one being built from interactions of two copies of Tyr_221_, whereas the other was built from a single copy of Tyr_221_ and the equivalent residue in the SpoA1 domain, Leu_141_ (Fig. 4B). Mutation of Tyr_221_ to Arg in both the SpoA2 domain of Spa33‐FL and within the two copies Spa33‐C prevented oligomerisation beyond assembly of the 1:2 complex (Fig. S6A). SEC‐MALS confirmed that higher order oligomerisation of this mutant was impeded in solution, whereas a control Ala mutation of Lys_235_, a surface exposed residue remote from the proposed dimer–dimer interface, showed no change in oligomerisation relative to wild‐type Spa33‐FL/C_2_ (Fig. S6B). Further mutation in the Y221R background of Leu_141_ to Ala (Fig. 5C and D) led to Spa33‐FL becoming entirely insoluble consistent with the behaviour of this protein in the absence of Spa33‐C. These data support the hypothesis that the interface required to build the 1:2 complex is the mixed interface involving the SpoA1‐Spa33‐FL/Spa33‐C chains while that required to assemble the elongated array involves the sequence identical SpoA2‐Spa33‐FL/Spa33‐C chains. In order to test the functional relevance of this higher order assembly, allelic exchange was used to introduce the Y221R point mutation and the Y221R/L141A double mutation within the *spa33* gene. Neither strain displayed secretion upon CR induction (Fig. S6C), indicating that the 1:2 complex alone is insufficient to support secretion.

Given the proximity of the Spa33_208‐293_ C‐terminus to the dimer–dimer interface in the crystal and the occlusion of this interaction site by the C‐terminal tag of YscQ‐C (Fig. S5B) (Bzymek *et al*., 2012), we reasoned that relatively small fusions to the C‐terminus of Spa33‐C would inhibit higher order assembly of the dimer. Indeed, addition of just an eight residue Strep‐tag to the C‐terminus of Spa33‐C_2_ (strep) within the 1:2 complex impeded further oligomerisation of this building block, with only up to four copies being observed in the native mass spectrum at very low abundance (Fig. S7A). Assembly was inhibited to an even greater extent for analogous thioredoxin (trx; 11.8 kDa) and maltose binding protein (mbp; 40.5 kDa) fusions, with the largest species observed being a dimer (Fig. S7B) and a monomer (Fig. S7C) of the 1:2 complex respectively. This is in agreement with *in vivo* data showing that a FliN‐YFP fusion is unable to complement for either swimming or swarming in an *E. coli ΔfliN* strain (Li and Sourjik, 2011).

The cryo‐EM reconstruction of the flagellar C‐ring from *S. typhimurium* has a continuous spiral of density 7.0 nm in diameter around the cytoplasmic edge of the C‐ring, which was previously suggested to fit a lock washer arrangement of SpoA domains (Thomas *et al*., 2006) such as that proposed here for Spa33‐FL/Spa33‐C_2_ (Fig. 5D). Indeed, positioning of the molecular model for the 1:2 complex within this density in the map (EMDB 1887) shows excellent agreement between the size and shape of Spa33‐FL/Spa33‐C_2_ and the density envelope (Fig. 5E). This fit not only provides further evidence for our models of Spa33‐FL/C_2_ and the high molecular weight oligomers it forms (Fig. 5A and D) but suggests a common mechanism of assembly for the NF‐ and flagellar C‐rings.

## Discussion

The subunit of the NF‐T3SS C‐ring is generally encoded by just one gene, whereas the flagellar C‐ring is largely comprised of two distinct but homologous proteins, FliM and FliN, and so it was difficult to assess the extent to which C‐ring structure and function differed between these systems from early work (Brown *et al*., 2005; Morita‐Ishihara *et al*., 2006). In this study, we show that *spa33* of the *S. flexneri* NF‐T3SS is alternatively translated *in vivo* to produce both the full‐length protein, Spa33‐FL, and also a construct comprising the C‐terminal third of the protein sequence, Spa33‐C. This is in agreement with recent findings from other NF‐T3SS, where two physiologically relevant translation products of *Salmonella* SPI‐2 *ssaQ* (Yu *et al*., 2011) and *Yersinia yscQ* (Bzymek *et al*., 2012) have also been identified, although the use of a GTG start codon instead of ATG seems so far to be a unique observation in *Shigella*. Indeed, the position of the Spa33‐C start site is conserved not only between these organisms, but with other as of yet uncharacterised homologues (Fig. S2), indicating that the expression of two products from one gene is a common strategy employed by NF‐T3SS. Furthermore, preliminary work from this study has hinted that *T. maritima fliY* could also be alternatively expressed in a similar manner to produce a FliN‐like protein, whereas several early studies showed that an alternative translation initiation site within *E. coli* FliN produces a fragment of the protein capable of supporting flagellar assembly (Irikura *et al*., 1993; Tang *et al*., 1995) (Fig. S2), suggesting that this mechanism could even be conserved in flagellar‐T3SS despite the presence of two protein‐coding genes.

Spa33‐FL and Spa33‐C form a complex *in vivo*, with both components being required for NF‐T3SS assembly and secretion, as previously observed for the *Yersinia* orthologues (Bzymek *et al*., 2012; Diepold *et al*., 2015). Furthermore, both YscQ‐FL and YscQ‐C are required for their localisation at the base of the NF‐T3SS (Diepold *et al*., 2015). Not only does this corroborate the pivotal role of these NF‐T3SS proteins for protein transport (Morita‐Ishihara *et al*., 2006; Lara‐Tejero *et al*., 2011), but also suggests that Spa33‐C is unlikely to just be acting as a chaperone for Spa33‐FL (Yu *et al*., 2011), with both proteins instead more likely to be integral structural components of the putative C‐ring in a similar manner to FliM and FliN. Indeed, our crystal structure of the SpoA2 domain of Spa33 revealed a highly intertwined homodimer that bears striking resemblance to both orthologous NF‐T3SS proteins (Fadouloglou *et al*., 2004; Bzymek *et al*., 2012) and flagellar FliN (Brown *et al*., 2005), with further characterisation revealing that Spa33‐C forms this observed dimer fold in solution with disordered N‐terminal extensions. Therefore, the sequence and structural homology between Spa33‐C and FliN would indicate they are likely to be playing a similar role *in vivo* within the C‐rings of NF‐ and flagellar‐T3SS.

Based on size and sequence similarities within their C‐terminal regions, Spa33‐FL and Spa33‐C, along with their SctQ orthologues, bear a striking resemblance to FliM and FliN respectively of the flagellar C‐ring. However, in this study, we present evidence that the full‐length SctQ proteins actually represent a fusion of FliM and FliN. This hypothesis is driven by two main observations. First, we have identified a second SpoA domain in the full‐length SctQ proteins, immediately N‐terminal to the well‐characterised C‐terminal domain. This SpoA1 domain shows a comparable level of sequence identity to the SpoA2 domain as the SpoA domains in FliM and FliN do to each other. Second, native MS has identified minimal Spa33‐FL/Spa33‐C_2_ and FliM/FliN_3_ complexes that are directly equivalent in terms of number and ratio of SpoA domains, i.e. one SpoA1 domain and three SpoA2 domains per complex. Specifically, we propose that one FliM (SpoA1) and one FliN (SpoA2) form an intermolecular heterodimer equivalent to an Spa33‐FL intramolecular SpoA1‐SpoA2 pseudodimer and that these then further associate with a FliN (SpoA2) homodimer and Spa33‐C (SpoA2) homodimer respectively.

Our results suggest that the structure formed by the N‐terminus of Spa33‐FL (Fig. 5A) is much smaller than the equivalent region of FliM (Fig. 5B) and therefore likely structurally distinct from its characterised CheC/CheX phosphatase fold (Park *et al*., 2006). Interestingly, based on sequence comparisons, the *P. syringae* NF‐T3SS C‐ring component HrcQ_A_ is likely to represent a hybrid protein, with the C‐terminus comprising a single SpoA1 domain like FliM (requiring the SpoA2 domain of HrcQ_B_ for dimer formation) and the N‐terminus a smaller domain similar in size to that of Spa33‐FL. Therefore, it is likely that the uncharacterised N‐terminal domain of Spa33‐FL orthologues could structurally and functionally differentiate NF‐C‐rings from their flagellar counterparts.

Previous modelling of the flagellar C‐ring has involved a FliM/FliN_4_ complex that was originally proposed on the basis of AUC measurements on the *T. maritima* FliM and FliN proteins (Brown *et al*., 2005). Our attempts to reproduce this complex using the same protein constructs led to native MS spectra that were complicated to interpret due to the presence of two different FliN species – the original sequence and a shorter fragment (FliN*). However, although they clearly showed a mixture of FliM/FliN complexes, we found no evidence for a 1:4 stoichiometry, with the dominant species being 1:3. A similar result was obtained using FliM complexed with full‐length FliY. We postulate that the complexity of the mixtures of species produced by coexpression of these proteins may give rise to averaged masses in sedimentation equilibrium AUC studies.

Several lines of evidence already present in the literature further support our molecular model for the 1:3 complex of FliM/FliN. Original estimates of protein copy numbers in the *S. typhimurium* C‐ring revealed ∼ 35 FliM and ∼ 111 FliN, in agreement with a 1:3 stoichiometry (Zhao *et al*., 1996). Similarly, a *S. typhimurium* mutant that only expresses a FliM–FliN fusion protein shows the propensity to form a limited number of fragmented C‐rings and could be complemented by the coexpression of FliN but not FliM (Kihara *et al*., 1996). This not only provides support for the association of FliN_2_ with a FliM/FliN heterodimer, but also directly mimics the SctQ proteins from the NF‐T3SS. Furthermore, this model also answers two questions that have been previously asked: (i) in the absence of dimerisation what fold does the orphan FliM SpoA domain assume, and (ii) why does the C‐terminal SpoA2 domain of Spa33‐FL not dimerise when the exact same sequence does in Spa33‐C? In both cases it has been suggested that the SpoA domain refolds into a monomeric, folded‐back structure in response to the presence of sequence at the N‐terminus (Sarkar *et al*., 2010; Bzymek *et al*., 2012). Our new model answers both of these questions using hetero‐dimerisation, removing the need for structural remodelling of a sequence that has only ever been observed in one conformation.

After initial submission of this manuscript, Notti *et al*. (2015) published a structure of the heterodimer formed by SpoA1/SpoA2 within *S. typhimurium* SpaO and provided *in vivo* data that suggest it is indeed formed intramolecularly. This dimer shows high structural homology to Spa33_208‐293_ (2.2 Å RMSD, 123 Cα atoms) (Fig. S8A), providing further evidence for the Spa33 SpoA1‐SpoA2 intramolecular dimer we have modelled and supporting our assertion that the SpoA1 domain is a conserved feature among NF‐T3SS C‐ring proteins. Furthermore, the crystal structure of a FliM(SpoA1)‐FliN(SpoA2) dimer shows the same fold (2.3 Å RMSD, 136 Cα atoms) (Fig. S8B) (Notti *et al*., 2015), indicating FliM and FliN are indeed able to heterodimerise as we have proposed.

Further analysis of the arrangement of Spa33 SpoA2 dimers within the crystal lattice revealed a dimer–dimer interface that is completely conserved in HrcQ_B_‐C (Fadouloglou *et al*., 2004) crystals, despite the proteins sharing only 16% sequence identity. This allowed us to propose a molecular model for the Spa33‐FL/C_2_ complex, whereby the internal pseudodimer of Spa33‐FL interacts with a homodimer of Spa33‐C in an analogous manner to the crystal packing, thereby forming an open lock washer structure.

In addition, construction of a homology model of *E. coli* FliM/FliN based on our molecular model for Spa33‐FL/C_2_ allowed a subset of the residue pairs whose Cys‐mutants previously led to cross‐linking of FliM/FliN (Sarkar *et al*., 2010) to be mapped (Fig. S9). This reveals that Asn_86_/Met_316_, Asn_72_/Met_260_, Asn_72_/Met_267_ and Asn_86_/Met_303_ are within close proximity within our model (Fig. S9) and therefore that this cross‐linking data is as consistent with a 1:3 complex of FliM/FliN as the previous (FliN)_4_‐FliM‐(FliN)_4_ model (Sarkar *et al*., 2010). Furthermore, given the intimate association of FliM and FliN within the heterodimer, it can also be envisaged how a subset of dramatic non‐conservative surface mutations of FliN were shown to prevent its interaction with FliM (Sarkar *et al*., 2010), suggesting that our 1:3 model for FliM and FliN assembly is entirely consistent with previous results. This FliM/FliN_3_ model in the context of earlier proposals for location of further C‐ring components (Park *et al*., 2006; Lee *et al*., 2010) leads to a molecular model for the entire flagellar C‐ring consistent with the density observed in the *S. typhimurium* reconstruction (Fig. S10).

In this study, we also show that this Spa33‐FL/C_2_ building block can undergo further controlled and directed oligomerisation *in vitro*, as would be required for this complex to form an ordered C‐ring *in vivo* similar to that found in the flagellar‐T3SS. These higher order oligomers are functionally important, as mutation of residues that destroy the interface abolishes substrate secretion. We therefore believe this represents the first observation of intermediates in assembly of the NF‐T3SS C‐ring. The open arrangement of the 1:2 complex enables the formation of a linear array via the conserved dimer‐dimer interface, allowing us to construct a molecular model for Spa33‐FL/C_2_ oligomers that shows good agreement with the shape of the high molecular weight species observed in the gas phase. Although our modelled and observed oligomers of Spa33‐FL/C_2_ are clearly elongated, formation of a closed ring structure would only require 10°–16° curvature per subunit to form a 22–34 member C‐ring, with the rotated dimer‐dimer interface observed in the FliN crystal (Fig. S5C) indicating that such flexibility in the interaction may be possible. Furthermore, the established interaction between Spa33 homologues and the basal body of the NF‐T3SS basal body (Morita‐Ishihara *et al*., 2006; Diepold *et al*., 2010; Barison *et al*., 2012) may be required to template C‐ring formation *in vivo*. However, positioning of our model into the cryo‐EM reconstruction of the *S. typhimurium* C‐ring (Thomas *et al*., 2006) reveals a striking correlation between the size and shape of Spa33‐FL/C_2_ and the spiral density at the cytoplasmic edge of the flagellar C‐ring, indicating this is likely to represent a physiologically relevant model for C‐ring assembly. This same spiral packing is observed within the crystal lattice of the newly released FliM/FliN heterodimer structure (Notti *et al*., 2015) and also provides a good fit to the EM density (Fig. S8C), indicating there is indeed conservation between the mode of assembly of NF‐ and flagellar C‐ring components.

Despite the wealth of other data suggesting an essential role for SctQ proteins at the base of the NF‐T3SS (Morita‐Ishihara *et al*., 2006; Diepold *et al*., 2010; Lara‐Tejero *et al*., 2011; Bzymek *et al*., 2012), it has remained controversial whether these systems have an organised substructure akin to the flagellar C‐ring. Recently, specific EM density for Spa33 in the expected position for a C‐ring was identified for the first time within the *S. flexneri* NF‐T3SS, although the tomograms showed six discrete Spa33‐FL/C_2_ ‘pods’ rather than a contiguous C‐ring (Hu *et al*., 2015). Although it is reasonable that the flagellar C‐ring structure could be remarkably different to account for its additional role in flagellar rotation and switching, the pods may also represent a subset of the Spa33‐FL/C_2_ population that is most stably associated with the sorting platform (presumably those subunits that are linked to the hexameric ATPase via SctL), perhaps comparable with the slow‐exchanging FliM population in the flagellar C‐ring (Delalez *et al*., 2010; Lele *et al*., 2012). Indeed, the observation that ∼ 22 copies of fluorescently‐labelled YscQ‐FL are localised at the base of the *Yersinia* NF‐T3SS still implies the formation of a larger structure (Diepold *et al*., 2015). Furthermore, the finding that subunits of both the NF‐ (Diepold *et al*., 2015) and flagellar (Delalez *et al*., 2010; Lele *et al*., 2012) C‐rings undergo rapid exchange *in vivo* is in agreement with the dynamic oligomerisation of Spa33‐FL/C_2_ observed in this study and indicates the putative NF‐T3SS C‐ring is unlikely to be a stable structure, perhaps explaining why it has been so hard to visualise in initial EM studies (Kawamoto *et al*., 2013; Kudryashev *et al*., 2013) and why more dynamic regions could plausibly still be missing in the most recent tomogram (Hu *et al*., 2015). Although further *in situ* characterisation of the NF‐T3SS C‐ring is clearly required, results from this study suggest that these systems have all the makings of a substructure highly similar to the flagellar C‐ring and that the molecular mechanisms of assembly of at least the subcomplexes of NF and flagellar C‐ring are fundamentally conserved.

## Experimental procedures

### DNA plasmids and mutagenesis

All constructs were created for this study (Table S1) either using the primers outlined (Table S2) or by Eurogentec. Unless otherwise stated, PCR products were subcloned into purified empty vectors digested with FastDigest enzymes (Fermentas) using the In‐Fusion PCR cloning system (Clontech). The Quikchange XL site‐directed mutagenesis kit (Stratagene) was used to create all subsequent point mutations, deletions and insertions. All constructs were verified by DNA sequencing.

### Recombinant protein expression and purification

Spa33‐FL/C_2_, Spa33‐FL/C_2_(Y221R), Spa33‐FL/C_2_(L141A/Y221R), Spa33_208‐293_, Spa33‐FL(CTD) and *T. maritima* FliM/FliN, FliM/FliY and FliM/FliN* were expressed from pET28b‐Spa33, pET28b‐Spa33(Y221R), pET28b‐Spa33(L141A/Y221R), pET28b‐Spa33_208‐293_, pETDuet‐Spa33‐FL(CTD), pETDuet‐FliM/FliN, pETDuet‐FliM/FliY and pETDuet‐FliM/FliN* plasmids (Table S1) respectively in *E. coli* B834 (DE3) grown in 4 × 1 l LB medium (Fisher Scientific UK). The Spa33‐FL(CTD)/C_2_, Spa33‐FL/Cstrep, Spa33‐FL/Ctrx and Spa33‐FL/Cmbp complexes were produced by coexpression from the pETDuet‐Spa33‐FL(CTD)/pRSFDuet‐Spa33‐C, pETDuet‐Spa33ΔRBS/pRSFDuet‐Spa33‐Cstrep, pETDuet‐Spa33ΔRBS/pRSFDuet‐Spa33‐Ctrx and pETDuet‐Spa33ΔRBS/pRSFDuet‐Spa33‐Cmbp plasmid pairs (Table S1) in a similar manner. Spa33‐C and Spa33‐CΔN were expressed as ^15^N‐labelled proteins from pET28b‐Spa33‐C and pET28b‐Spa33‐CΔN plasmids (Table S1) respectively in *E. coli* BL21 (DE3) grown in 2 × 1 l ^15^N‐labelled M9 minimal medium. Cells were generally grown at 37°C until A_600 nm_ of ∼ 0.6 was reached, and then protein expression was induced overnight at 21°C with 1 mM IPTG. Cells were lysed in buffer containing 50 mM Tris‐HCl pH7.5, 500 mM NaCl, 1 mM DTT and a Protease Inhibitor tablet (Pierce) using an Emulsiflex‐C5 Homogeniser (GC Technologies) and the His‐tagged protein within the clarified lysate extracted using a 5 ml Ni^2+^‐NTA superflow cartridge (Qiagen). Tags were removed from some constructs overnight during dialysis at 4°C, using thrombin (Amersham Biosciences) for Spa33‐C and Spa33‐CΔN and carboxypeptidase A (Sigma Aldrich) for Spa33_208‐293_. SEC was then carried out using a HiLoad 16/60 Superdex 200 pg (GE Healthcare) column equilibrated in 20 mM Tris‐HCl pH7.5, 150 mM NaCl, 1 mM TCEP.

### Western blotting

Rabbit anti‐Spa33 polyclonal antibodies were raised against untagged Spa33_208‐293_ and purified using a Spa33_208‐293_ affinity column by Eurogentec. Samples were separated via SDS‐PAGE and transferred to a Hybond‐P membrane (GE Healthcare) using a trans‐blot semi‐dry transfer cell (Bio‐Rad) for 1 h at 22 V. Membranes were blocked overnight at 4°C in 2% w/v milk, 1× PBS and 0.1% v/v Tween. α‐Spa33 was used as the primary antibody at 1:1000–1:50 000 dilutions, whereas a HRP‐conjugated anti‐rabbit IgG secondary antibody (Promega) was used at 1:2500 dilution. Membranes were developed with an ECL Western Blotting System (GE Healthcare) and exposed to Amersham Hyperfilm ECL (GE Healthcare).

### Crystallisation and structure determination of Spa33_208‐293_

The 10 mg ml^−1^ Spa33_208‐293_ was crystallised at 21°C by the vapour‐diffusion sitting‐drop method in 400 nl drops at a 1:1 ratio with 10% (v/v) isopropanol, 0.1 M Na HEPES pH7.0, 10% (w/v) PEG 4000 using an OryxNano Crystallisation Robot (Douglas Instruments). Crystals were cryo‐protected with 1:4 (v/v) ethylene glycol : mother liquor and flash‐frozen in liquid N_2_. Diffraction images were collected at beamline I04 of the Diamond Light Source (Oxfordshire, UK) and processed as P2_1_2_1_2_1_ using the Xia2 pipeline in the 3dii mode (Winter, 2010). Initial phases were calculated following molecular replacement using Phaser (McCoy *et al*., 2007) with an ensemble of *T. maritima* FliN (pdb id 1YAB) and *P. syringae* HrcQ_B_‐C (pdb id 1O9Y) chainsaw (Stein, 2008) models, trimmed to remove regions of poor structural alignment. The model was rebuilt and refined iteratively using Coot (Emsley *et al*., 2010) and autoBUSTER (Blanc *et al*., 2004) or Phenix (Adams *et al*., 2010) but R_free_ stalled around 30% and the maps contained almost no ordered solvent despite the 2.3 Å resolution. Running the data through Xtriage suggested the data may be twinned and suggested reprocessing in P2_1_ which was carried out using Xia2 in the 3daii mode (Table 2). Two copies of the Spa33_208‐293_ dimer were placed using Phaser and refined in Phenix using the twin law (h, ‐k, ‐l). The model was rebuilt and refined iteratively using Coot and Phenix. Protein chemistry was validated using Molprobity (Davis *et al*., 2007) and the final model visualised with PyMol (Schrödinger). The co‐ordinates for Spa33_208‐293_ have been deposited in the Protein Data Bank as entry pdb id 4TT9.

### NMR spectroscopy

^15^N‐labelled Spa33‐C and Spa33‐CΔN were dialysed into 20 mM Tris‐HCl pH7.5, 50 mM NaCl to improve spectral quality. All samples were diluted to 100 μM protein and supplemented with 5% v/v D_2_O. ^1^H,^15^N‐HSQC spectra were recorded at 25°C on a Bruker Avance II 500 MHz spectrometer. Spectra were processed using TopSpin (Bruker) and analysed with Sparky (Goddard and Kneller, 2001).

### Native MS

Samples were buffer‐exchanged in 200 mM ammonium acetate pH 7.5 using benchtop size‐exclusion columns (Micro‐Biospin 6, Bio‐Rad) and directly loaded on borosilicate needles prepared in‐house (Hernandez and Robinson, 2007). All the experiments were performed on a hybrid quadrupole ion mobility time‐of‐flight mass spectrometer (Synapt HDMS, Waters) modified for the transmission of high molecular weight complexes and for the determination of absolute collision cross‐sections without any prior calibration (Bush *et al*., 2010). The parameters used for the IM‐MS analysis were the following: 1.5 kV, 20 V, 0.8 V and 10 V for the capillary, sample cone, extraction cone and trap cell voltages. Drift times were recorded with drift voltages ranging from 50 to 100 V with 10 V increments. Gas pressures were set at 6.7e‐3, 6.7e‐2, 4.6 and 2.4e‐6 bar in the source, quadrupole, trap (argon at 5 ml min^−1^) and mobility cell (helium at 50 ml min^−1^) respectively. The data were recorded and analysed with MassLynx and Driftscope softwares (Waters), and the spectra were calibrated using a 100 mg ml^−1^ solution of cesium iodide. Theoretical collision cross‐sections of pdb files and generated models were measured using the scaled projection approximation method (Benesch and Ruotolo, 2011). When calculating CCS values for assemblies for which only a partial atomic structure was available, the mass for missing atoms m was taken into account through incrementing CCS measures by (n*m)**(2/3), where n is the number of subunits in the assembly.

### MALS

SEC was performed on Superdex 200 10/300 column (GE Healthcare) equilibrated in 20 mM Tris‐HCl pH7.5, 150 mM NaCl. 100 μl of protein was injected at increasing concentrations and eluted at 0.4 ml min^−1^. The column was followed in line by a Dawn Heleos‐II light scattering detector (Wyatt Technologies) and an Optilab‐Rex refractive index monitor (Wyatt Technologies). Molecular mass calculations were performed using ASTRA 6.1.1.17 (Wyatt Technologies) assuming a dn/dc value of 0.186 ml g^−1^.

### Construction of *S* *. flexneri* strains

Approximately 1 kb of DNA upstream and downstream of *spa33* was amplified from *S. flexneri* M90T (M90T; Table 1) virulence plasmid and joined to each side of an amplified *sacB‐kanR* cassette (Blomfield *et al*., 1991) via the Gibson Assembly reaction, according to manufacturer guidelines (New England Biolabs) and using the primers outlined (Table S2). The resulting linear construct was integrated into the virulence plasmid using the λ Red system expressed from the pKD46 plasmid (Table S1) (Datsenko and Wanner, 2000) and selected on kanamycin to produce an intermediate strain for allelic exchange (GMCT113; Table 1).

Variant *spa33* sequences were amplified with mutagenic primers (Table S2) and cloned into a derivative of the temperature‐sensitive vector pKO3 (Link *et al*., 1997) lacking its own copy of the *sacB* gene. These constructs (Table S1) were used to carry out the allelic exchange (Blomfield *et al*., 1991). Briefly, vectors were electroporated into GMCT113 and transformants selected on chloramphenicol at 30°C. The resulting vector‐containing cells were passaged in antibiotic‐free liquid medium for 3 h at 42°C and plated on chloramphenicol at 42°C to obtain vector integrates in the virulence plasmid. Subsequently, cells were grown again in antibiotic‐free liquid medium for 3 h at 42°C and then plated at 30°C on media containing 10% sucrose but lacking NaCl. Cells that formed colonies on sucrose had successfully excised the *sacB‐kanR* cassette. These sucrose‐resistant colonies were screened for sensitivity to kanamycin and chloramphenicol, confirming both curing of the vector and excision of the *kanR* marker. Finally, the *spa33* gene and flanking regions were sequenced to confirm the presence of the desired allele with no further mutations.

### CR induction assay

*Shigella flexneri* strains (Table 1) were grown in TCSB at 37°C until A_600nm_ of ∼ 1.0, at which point samples were taken for Western blotting of whole cell lysate. The CR induction assay was then performed as described previously (Kenjale *et al*., 2005). Twenty microlitres of bacterial supernatant was separated by SDS‐PAGE and silver‐stained with the SilverXpress kit (Invitrogen).

## Supporting information

Supporting information
