# Supplementary material for: Characterisation of Shigella Spa33 and Thermotoga FliM/N reveals a new model for C‐ring assembly in T3SS
Source: Mol Microbiol. 2015 Dec 23;99(4):749–66. doi: 10.1111/mmi.13267 (PMC4832279; doi:10.1111/mmi.13267)
Supplement: Supplementary file 1 — Supporting information [file MMI-99-749-s001.docx]

**Supplementary Figures and Tables for:**

**Characterisation of *Shigella* Spa33 and *Thermotoga* FliM/N reveals a new model for C-ring assembly in T3SS**

Melanie A. McDowell^1,3^, Julien Marcoux^2,4^, Gareth McVicker^1^, Steven Johnson^1^, Yu Hang Fong^1^, Rebecca Stevens^1^, Lesley A. Bowman^1^, Matteo T. Degiacomi^2^, Jun Yan^2^, Adam Wise^1^, Miriam Friede^1^, Justin L. P. Benesch^2^, Janet E. Deane^1,5^, Christoph M. Tang^1^, Carol V. Robinson^2^ and Susan M. Lea^1*^

^1^Sir William Dunn School of Pathology, University of Oxford, Oxford, UK.

### ^2^Department of Chemistry, University of Oxford, Oxford, UK.

### ^*^Corresponding author: [susan.lea@path.ox.ac.uk](mailto:susan.lea@path.ox.ac.uk), +44 1865 275181

### Current addresses:

### ^3^Biochemistry Centre (BZH), University of Heidelberg, Im Neuenheimer Feld 328, Heidelberg, Germany.

^4^Institute of Pharmacology and Structural Biology (IPBS), CNRS, UMR 5089, 205 Route de Narbonne, Toulouse, France

### ^5^Cambridge Institute for Medical Research, Wellcome Trust/MRC Bldg., Addenbrooke's Hospital, Hills Road, Cambridge, UK

### Running title: Uniform C-ring assembly by NF- and flagellar-T3SS

### Key words: T3SS, C-ring, pathogenesis, *Shigella*, structural biology, protein interactions


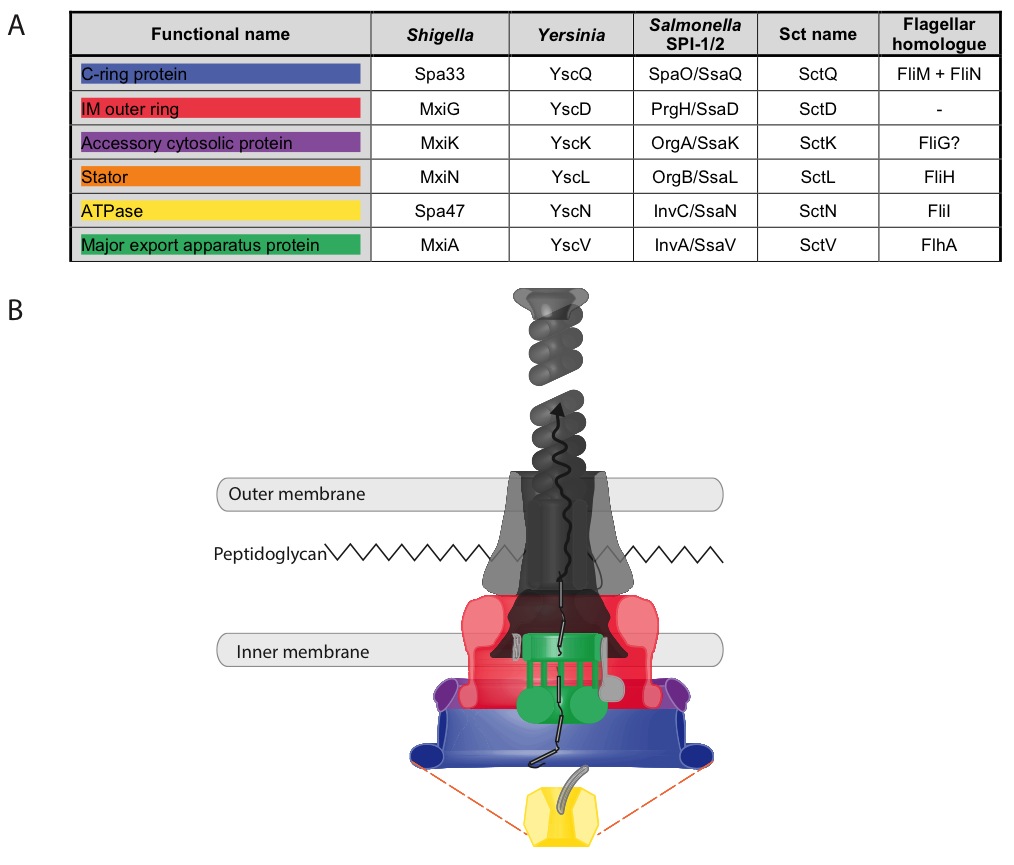


***Fig. S1 – Schematic highlighting components of the NF- and flagellar-T3SS mentioned in this study***

**A** Species specific names from the *Shigella*, *Yersinia*, *Salmonella* SPI-1/2 and flagellar-T3SS along with the unified Sct name ([Abrusci *et al.*, 2014](#_ENREF_1)) are given for the C-ring protein(s) (blue), IM outer ring protein (red), accessory cytosolic protein (purple), stator (orange), ATPase (yellow) and major export apparatus (green).

**B** The position of these components within the *Shigella* T3SS is also shown.

***
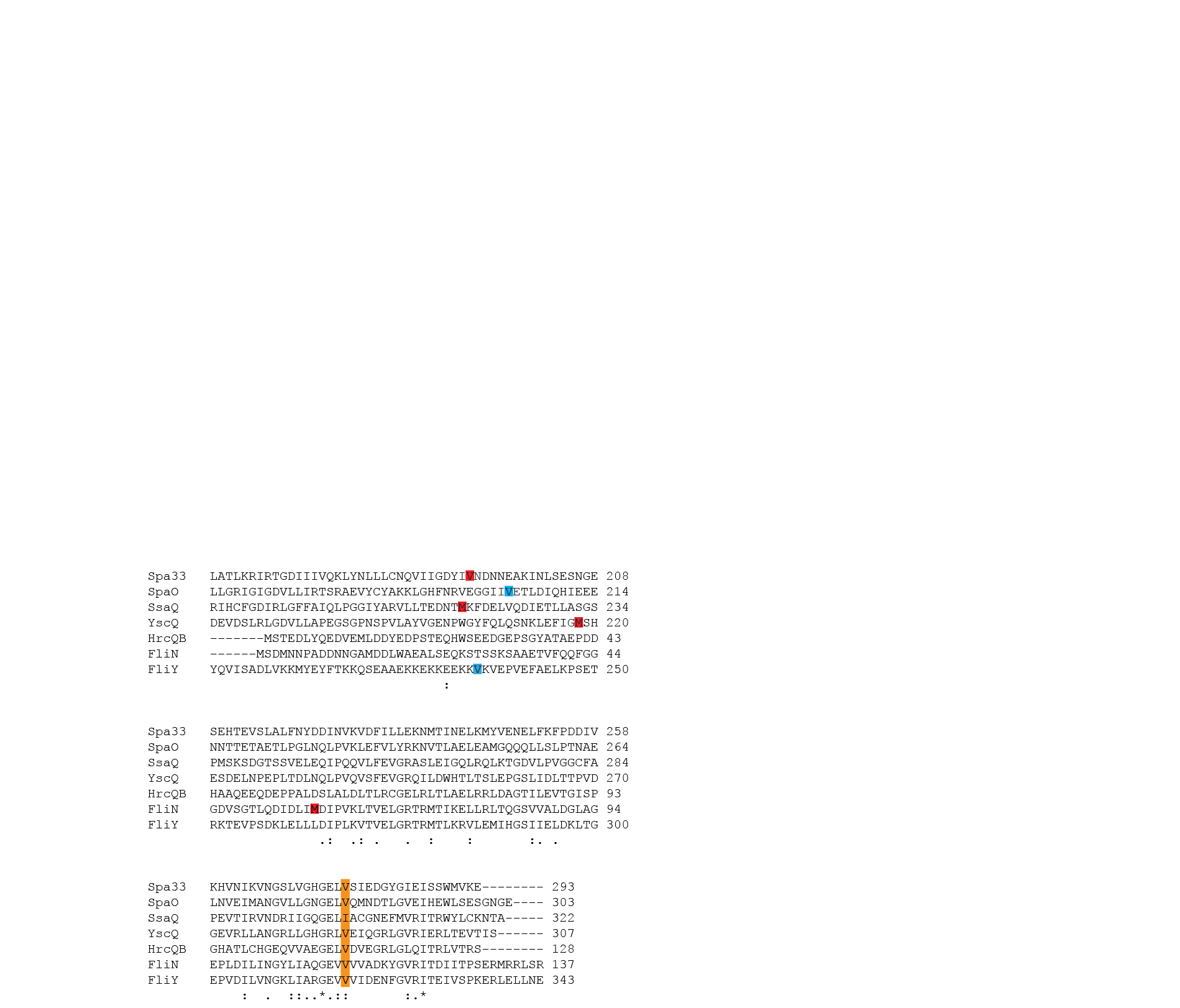
Fig S2 - Sequence alignment of Spa33-C with NF- and flagellar-T3SS orthologues***

Multiple sequence alignment of the C-terminus of *S. flexneri* Spa33 with the equivalent regions of *S. typhimurium* SPI-1 SpaO, *S. typhimurium* SPI-2 SsaQ, *Y. pseudotuberculosis* YscQ, *P. syringae* HrcQ_B_, *E. coli* FliN and *T. maritima* FliY performed using ClustalW2 ([Chenna *et al.*, 2003](#_ENREF_15)), with identical, conserved and semi-conserved residues being indicated by asterisks, colons and full-stops respectively. Sequence numbers are shown relative to the full-length protein. Alternative translation start sites that have either been experimentally demonstrated or predicted are highlighted in red and blue respectively. The conserved Val/Ile residues predicted to be involved in binding FliH homologues are highlighted in orange.


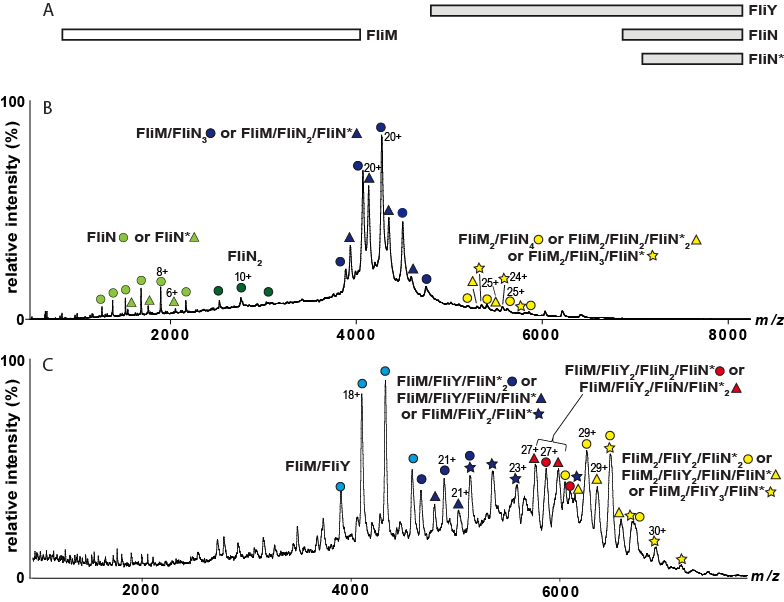


***Figure S3 – T. maritima FliM/FliN and FliM/FliY complexes are heterogeneous and sample a range of oligomeric states***

**A** Schematic of the species observed in the spectra shown in **B** and **C**. Size and sequence overlap of FliN and FliN* are shown relative to *T. maritima* FliY.

**B** Mass spectrum for *T. maritima* FliM/FliN complexes obtained under non-denaturing conditions, showing multimeric assemblies comprising various ratios of FliN and FliN* (light green). FliN dimers (dark green) and FliM/FliN complexes with either a 1:3 (dark blue; FliM/FliN_3_ or FliM/FliN_2_/FliN*) or 2:4 (yellow; FliM_2_/FliN_4_, FliM_2_/FliN_2_/FliN*_2_ or FliM_2_/FliN_3_/FliN*) stoichiometry are distinguishable. For each assigned complex, the charge state of the most intense peak is shown and a comparison of experimental and theoretical molecular mass is given in Table 3.

**C** Mass spectrum for *T. maritima* FliM/FliY complexes obtained under non-denaturing conditions, showing multimeric assemblies comprising various ratios of FliY, FliN and FliN*. FliM/FliY complexes with either a 1:1 (light blue), 1:3 (dark blue; FliM/FliY/FliN*_2_, FliM/FliY/FliN/FliN* or FliM/FliY_2_/FliN*), 1:5 (red; FliM/FliY_2_/FliN_2_/FliN* or FliM/FliY_2_/FliN/FliN*_2_) or 2:4 (yellow; FliM_2_/FliY_2_/FliN*_2_, FliM_2_/FliY_2_/FliN/FliN* or FliM_2_/FliY_3_/FliN*) stoichiometry are distinguishable. For each assigned complex, the charge state of the most intense peak is shown and a comparison of experimental and theoretical molecular mass is given in Table 3.

***
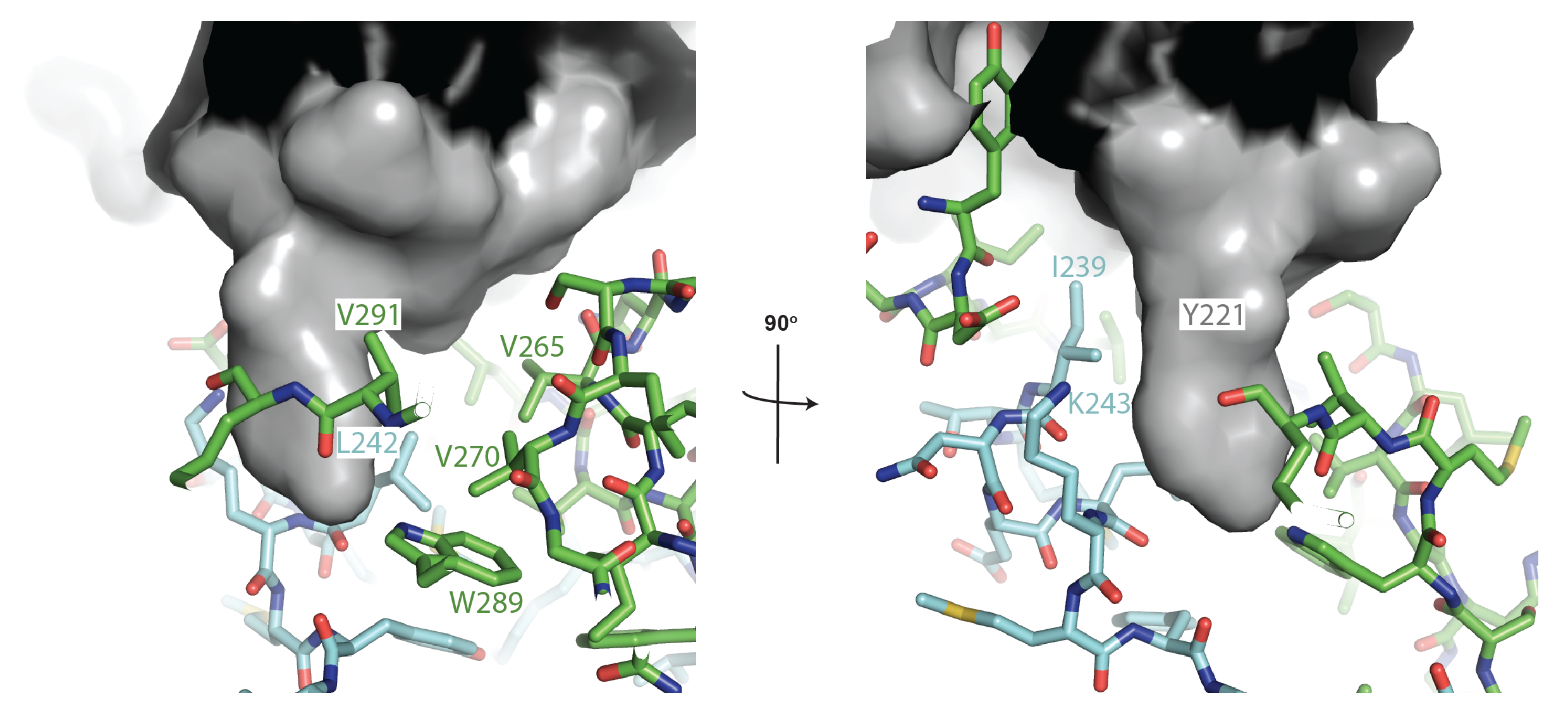
***

***Figure S4 – Detailed view of the dimer-dimer interface within the Spa33_208-293_ crystal lattice***

Two copies of the Spa33_208-293_ dimer extracted directly from the crystal lattice showing a zoomed view of the dimer-dimer interface. One copy of the dimer is shown as a surface representation, with chain A (black) and chain B (grey) differentiated and the key interacting residue Tyr_221_ labelled. The second copy is shown by a stick representation, with chain A (green) and chain B (light blue) differentiated and the residues lining the hydrophobic pocket labelled.

***
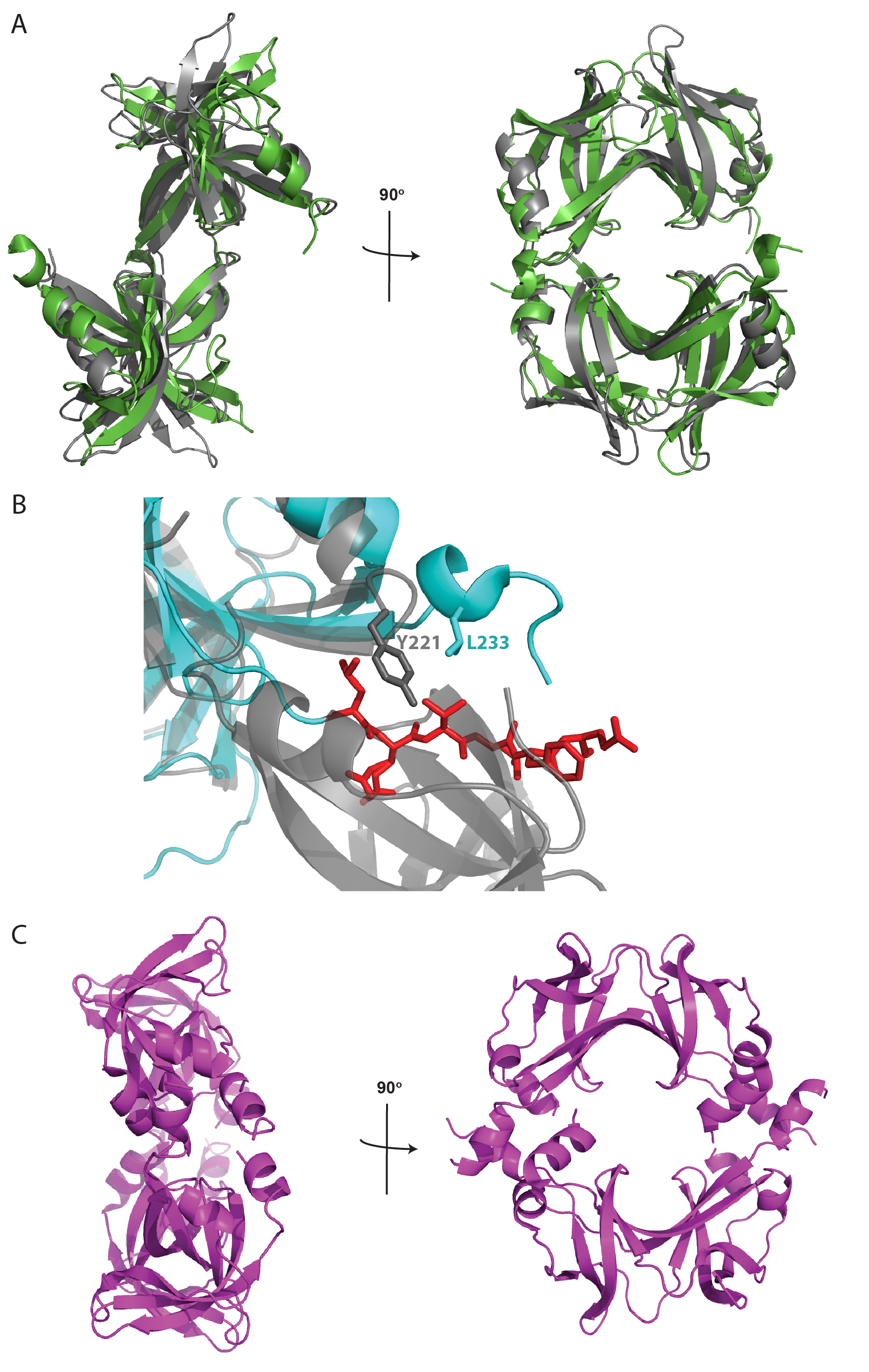
***

***Figure S5 – Comparison of packing within the crystal lattices of Spa33_208-293_ and homologous structures***

**A** Superimposition of two copies each of the Spa33_208-293_ dimer (grey) and HrcQ_B_-C dimer (green; pdb id 1O9Y) extracted directly from their crystal lattices, highlighting the conserved dimer-dimer interface.

**B** Superimposition of the Spa33_208-293_ dimer (grey) and YscQ-C dimer (turquoise; pdb id 3UEP), showing a second copy of Spa33_208-293_ extracted directly from the crystal lattice and a zoomed view of the resulting dimer-dimer interface. The non-native LEVLFQ tag stub of YscQ-C (red) interacts with L233, the equivalent residue to Y221 in Spa33_208-293_, and clashes with the second copy of Spa33_208-293_, demonstrating how an interaction via the same dimer-dimer interface was unable to form in the YscQ-C crystal lattice.

**C** Two copies of the FliN dimer (magenta; pdb id 1YAP) extracted directly from their crystal lattices, highlighting the conserved dimer-dimer interface.


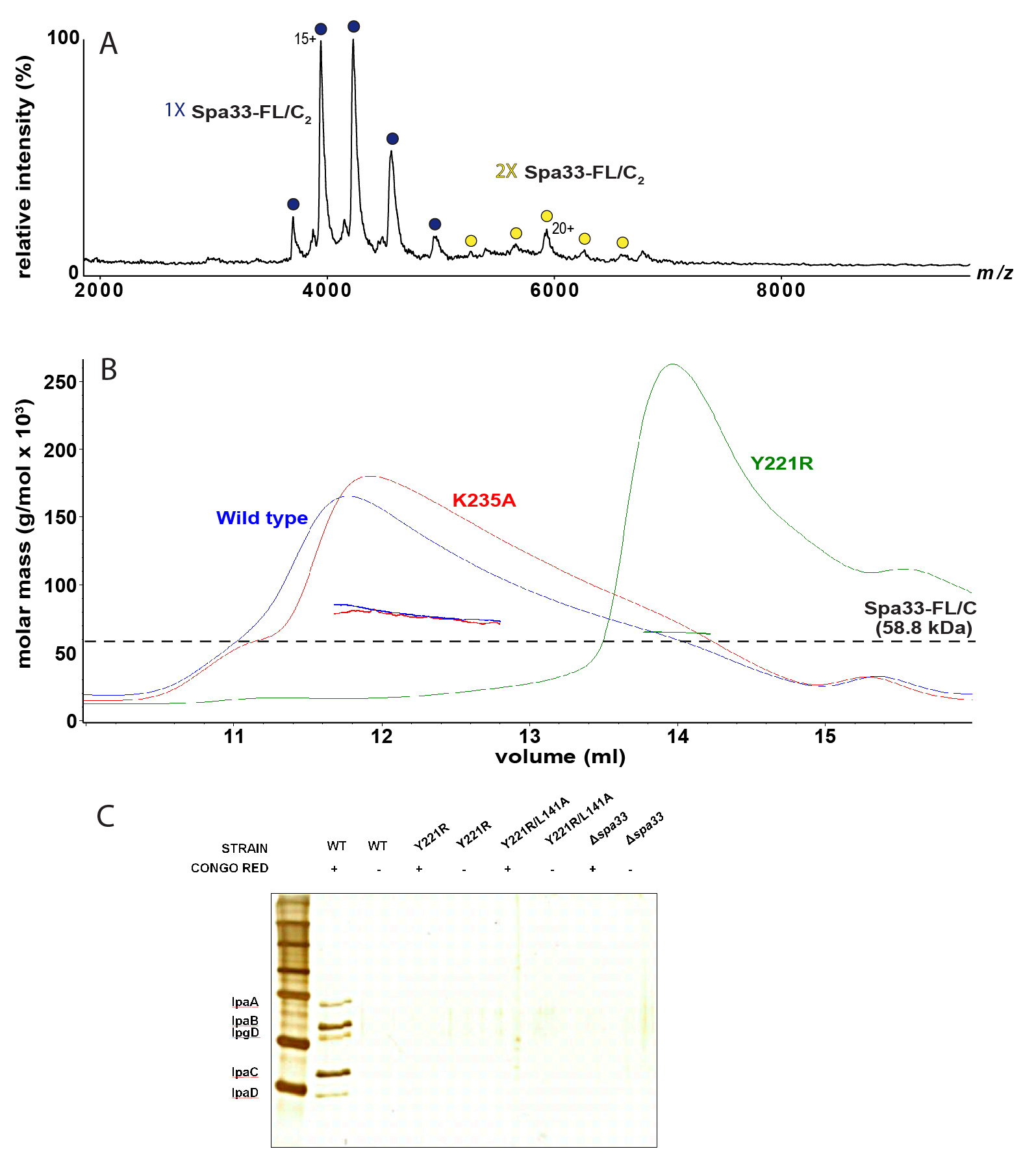


***Figure S6 – Mutation of Tyr_221_ at the conserved dimer-dimer interface disrupts higher order oligomerisation of Spa33-FL/C_2_***

**A** Native mass spectrum of His-Spa33-FL/Spa33-C complexes containing a Y221R mutation in both proteins. Monomeric (dark blue) and low levels of dimeric (yellow) Spa33-FL/C_2_ can be observed. For each assigned complex, the charge state of the most intense peak is shown and a comparison of experimental and theoretical molecular mass is given in Table 3. A parallel spectrum of the wild type protein at an the same concentration was collected (data not shown) and resembles Fig. 3A.

**B** Analysis of the oligomeric state of His-Spa33-FL/Spa33-C complexes containing no mutation (wild type; blue), a Y221R mutation (green) or a control K235A mutation (red) by SEC and in-line MALS. The left axis represents the molecular mass at any given point of the chromatogram. Elution profiles are shown for the main elution peak for 30 μM each complex and the expected molar mass for Spa33-FL/C_2_ is marked.

**C** CR induction assay for WT, Y221R, Y221R/L141A and *Δspa33* strains of *S. flexneri* generated by allelic exchange. Cell cultures were supplemented with 0.2 mg/ml CR and samples taken from the supernatant were separated on a 10% SDS-PA gel and silver-stained. The position of early effectors within the characteristic gel profile are shown.


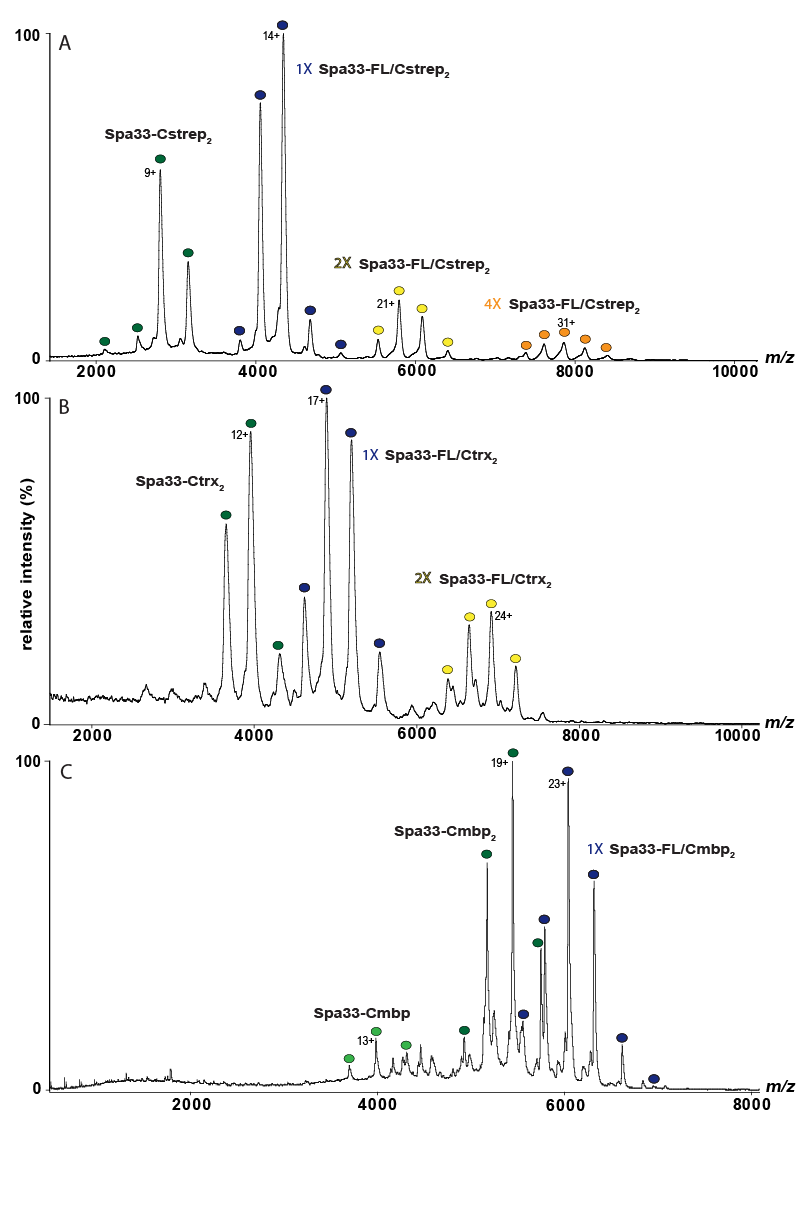


***Figure S7 – Fusion of a C-terminal tag to Spa33-C_2_ disrupts higher order assembly of the 1:2 complex***

Mass spectra for Spa33-FL/Spa33-C complexes with either a **A** strep, **B** thioredoxin (trx) or **C** maltose binding protein (mbp) C-terminal fusion to Spa33-C obtained under non-denaturing conditions. Monomers (light green) and dimers (dark green) of Spa33-C are observed, along with monomers (dark blue), dimers (yellow) and tetramers (orange) of the Spa33-FL/C_2_ complex. For each assigned species, the charge state of the most intense peak is shown and a comparison of experimental and theoretical molecular mass is given in Table 3.

***
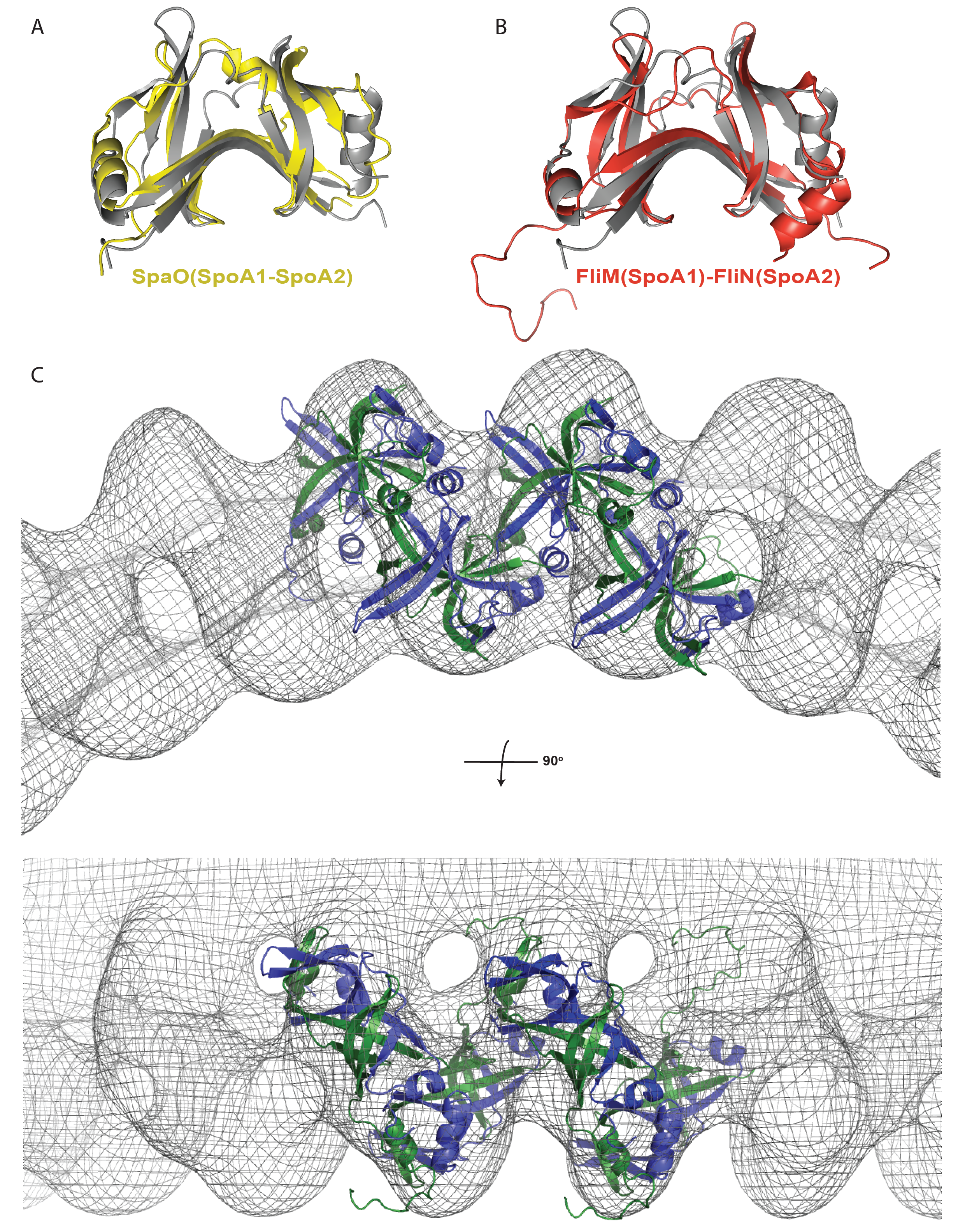
***

***Figure S8 – Recent crystal structures of S. typhimurium SpaO (SpoA1-SpoA2) and FliM(SpoA1)-FliN(SpoA2) corroborate our molecular models for C-ring assembly***

Superimposition of the Spa33_208-293_ (grey) with the structures of *S. typhimurium* **A** SpaO(SpoA1-SpoA2) (yellow; pdb id 4YX5) and **B** FliM(SpoA1)-FliN(SpoA2) (red; pdb id 4YXC).

**C** The crystal packing of FliM-FliN heterodimers shows a good agreement with the cryo-EM map of the flagellar C-ring. A spiral of dimers interacting via the conserved dimer-dimer interface was directly extracted from the crystal lattice and manually positioned in the density corresponding to the cytoplasmic edge of the C-ring in the C34 *S. typhimurium* EM map contoured to contoured to 1σ ([Thomas *et al.*, 2006](#_ENREF_57))(EMDB 1887). SpoA1 of FliM is coloured green, whilst SpoA2 of FliN is coloured blue.


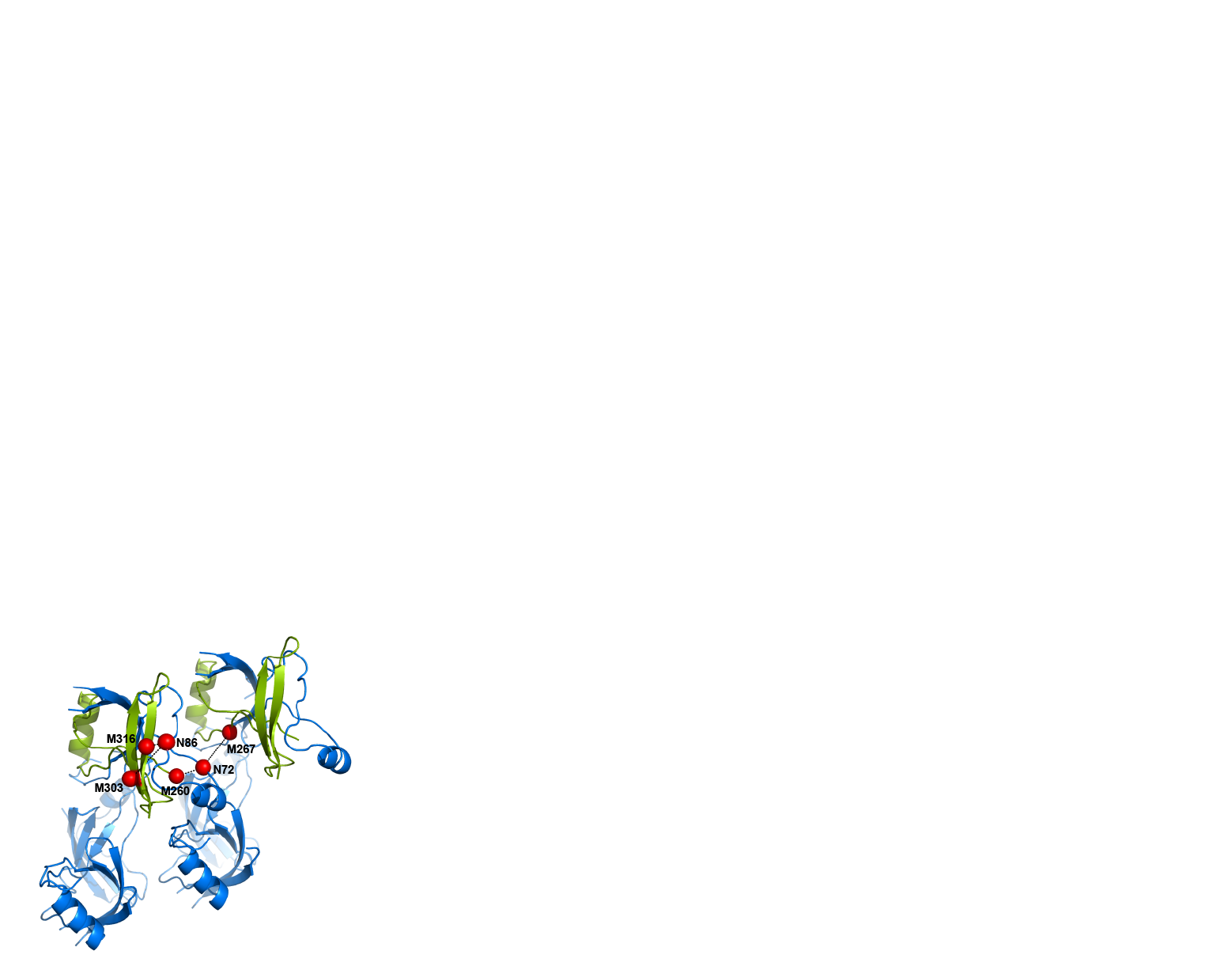


***Figure S9 – The formation of 1:3 complexes by FliM/FliN is consistent with published cross-linking data***

A SCWRL homology model for an assembly of the 1:3 complex of *E. coli* FliM (green) and FliN (blue) is shown, constructed using the sequence alignment with *S. flexneri* Spa33 shown in Figure 4B and the structural coordinates for the model shown in Figure 5D. Residues 72 and 86 within FliN and residues 260, 267, 303 and 316 within FliM are represented by a red sphere for their C^α^ atom and the cross-links previously identified for cysteine mutants of these residues ([Sarkar *et al.*, 2010](#_ENREF_53)) are represented by dashed black lines. As for each pairwise combination of FliM and FliN residues there are several possible intermolecular distances within the model, the shortest distance has been chosen here to represent the likely cross-link. Asn_134_/Met_310_, Asn_76_/Met_327_, Asn_76_/Met_328_, Asn_134_/Met_265_ and Asn_134_/Met_267_ cross-links are unable to be represented as they involve residues not found within our homology model for *E. coli* FliM/FliN.


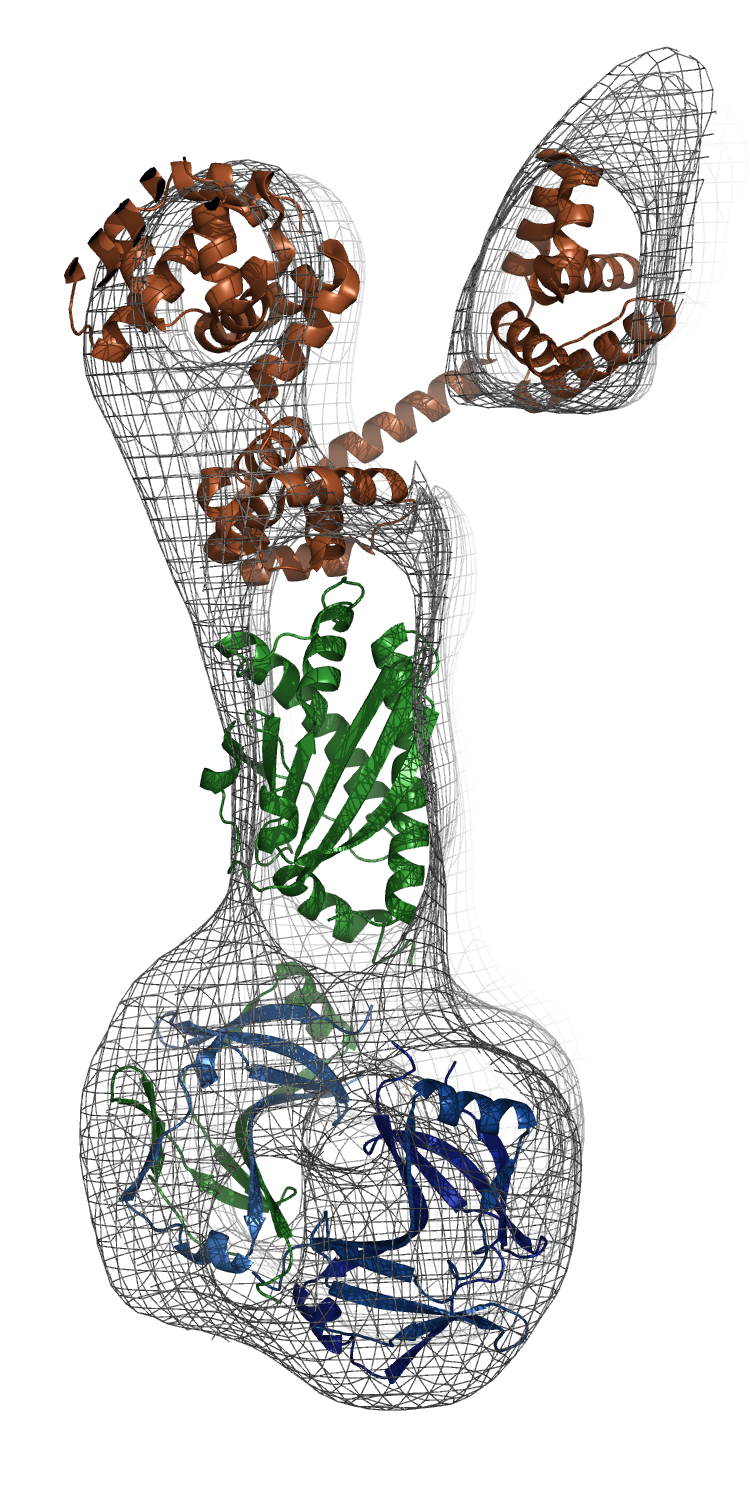


***Figure S10*** - ***Model demonstrating the likely arrangement of C-ring proteins as docked into the clockwise-locked cryo-EM map (EMD-1887).***

The FliM/FliN_3_ model (blue/green) from this study is docked into the spiral density at the bottom as in Figure 5. The complex of the middle domain of FliM (green) and the middle domain of FliG (brown) (pdb id 3SOH) is placed in the density above the spiral, and the remaining two domains of FliG (brown) (pdb id 3HJL) placed in the prominent densities above the FliM ring. Coordinates were manually positioned by hand in Coot and are intended to be illustrative rather than absolute relative positions.

**Table S1 – Plasmids created in this study**

| **Name** | **Description and source** |
| --- | --- |
| pET28b-Spa33 | Full-length *spa33* amplified from the virulence plasmid (Table S2) cloned between NdeI and BamHI of pET28b (Novagen), Kan^r^ |
| pET28b-Spa33(Y221R) | Y221R mutation introduced into *spa33* within pET28b-Spa33 by Quikchange mutagenesis (Table S2), Kan^r^ |
| pET28b-Spa33(L141A/Y221R) | L141A mutation introduced into *spa33(Y221R)* within pET28b-Spa33(Y221R) by Quikchange mutagenesis (Table S2), Kan^r^ |
| pET28b-Spa33_208-293_ | Residues 208-293 of *spa33* amplified from pET28b-Spa33 (Table S2) cloned between NcoI and XhoI of pET28b (Novagen), Kan^r^ |
| pET28b-Spa33-C | Residues 193-293 of *spa33* cloned amplified from pET28b-Spa33 (Table S2) between NdeI and BamHI of pET28b (Novagen), Kan^r^ |
| pET28b-Spa33-CΔN | Residues 208-219 of *spa33* deleted from pET28b-Spa33_208-293_ by Quikchange mutagenesis (Table S2), Kan^r^ |
| pETDuet-Spa33ΔRBS | Full-length *spa33* gene with a N-terminal MGSSHHHHHHSSGLVPRGSH His-tag amplified from pET28b-Spa33 (Table S2) cloned between NcoI and BamHI of pETDuet-1 (Novagen). GGGGA→GGTGA mutation in RBS and GTG→GTC mutation in alternative start codon introduced by Quikchange mutagenesis (Table S2), Amp^r^ |
| pETDuet-Spa33-FL(CTD) | Residues 1-131 of *spa33* deleted from pETDuet-Spa33ΔRBS by Quikchange mutagenesis (Table S2), Amp^t^ |
| pRSFDuet-Spa33-C | Residues 194-293 of *spa33* amplified from pET28b-Spa33 (Table S2) cloned between NcoI and BamHI of pRSFDuet-1 (Novagen), Kan^r^ |
| pRSFDuet-Spa33-Cstrep | C-terminal WSHPQFEK Strep-tag inserted downstream of *spa33-C* within the pRSFDuet-Spa33-C by Quikchange mutagenesis (Table S2), Kan^r^ |
| pRSFDuet-Spa33-Ctrx | Residues 2-109 of *E. coli thioredoxin* with a N-terminal GSAM linker amplified (Table S2) and cloned between BamHI and EcoRI of pRSFDuet-Spa33-C. The stop codon at the C-terminus of Spa33-C was subsequently removed by Quikchange mutagenesis (Table S2), Kan^r^ |
| pRSFDuet-Spa33-Cmbp | Residues 29-391 of *E. coli malE* with a N-terminal GSAM linker amplified (Table S2) and cloned between BamHI and HindIII of pRSFDuet-Spa33-C. The stop codon at the C-terminus of Spa33-C was subsequently removed by Quikchange mutagenesis (Table S2), Kan^r^ |
| pETDuet-FliM/N | Full-length *T. maritima* *fliM* with a N-terminal MGSSHHHHHHSSGLVPRGSH His-tag synthesised and cloned between NcoI and BamHI of pETDuet-1 (Novagen) by Eurogentec Ltd. Residues 212-343 of *T. maritima fliY* synthesised and cloned between NdeI and XhoI by Eurogentec Ltd, Amp^r^ |
| pETDuet-FliM/Y | Full-length *T. maritima* *fliM* with a N-terminal MGSSHHHHHHSSGLVPRGSH His-tag synthesised and cloned between NcoI and BamHI of pETDuet-1 (Novagen) by Eurogentec Ltd. Full-length *T. maritima fliY* amplified from pET28b-FliY (Table S2) cloned between NdeI and XhoI, Amp^r^ |
| pETDuet-FliM/N* | Residues 212-234 deleted from *fliY* within pETDuet-FliM/N by Quikchange mutagenesis (Table S2), Amp^r^ |
| pIB279 | ([Blomfield *et al.*, 1991](#_ENREF_9)); template for amplification of *sacB-kanR* fragment (Table S2). |
| pKD46 | ([Datsenko & Wanner, 2000](#_ENREF_17)); plasmid used for expression of the λ Red system. |
| pGM133 | pKO3 (*sacB^-^*) derivative with *spa33ΔFL* fragments inserted via the Gibson Assembly reaction (Table S2). |
| pGM134 | pKO3 (*sacB^-^*) derivative with *spa33ΔC* fragments inserted via the Gibson Assembly reaction (Table S2). |
| pGM135 | pKO3 (*sacB^-^*) derivative with *Δspa33* fragments inserted via the Gibson Assembly reaction (Table S2). |
| pGM136 | pKO3 (*sacB^-^*) derivative with *spa33ΔFL/ΔC* fragments inserted via the Gibson Assembly reaction (Table S2). |

**Table S2 – Primers used in this study**

| **Primer Description** | **Forward sequence (5’→3’)** | **Reverse sequence (5’→3’)** |
| --- | --- | --- |
| pET28b-Spa33 (cloning) | GCCGCGCGGCAGCCATATGAATGCTAAGAATTAAACATTTTGACGCTAACG | CGGAGCTCGAATTCGGATCCTTACTCCTTTACCATCCAAGAACTAATCTCG |
| pET28b-Spa33(Y221R) (mutagenesis) | ACACACAGAAGTTTCTTTGGCATTATTCAATCGTGATGATATCAATGTAAAAGTGG | CCACTTTTACATTGATATCATCACGATTGAATAATGCCAAAGAAACTTCTGTGTGT |
| pET28b-Spa33(L141A/Y221R) (mutagenesis) | AGATGCCGGTATTACGTAATCAAGTTTCTGCTGACTTATTGCATCATC | GATGATGCAATAAGTCAGCAGAAACTTGATTACGTAATACCGGCATCT |
| pET28b-Spa33_208-293_ (cloning) | TAAGAAGGAGATATACCATGGAGTCAGAACACACAGAAGTTTC | GTGGTGGTGGTGGTGCTCGAGCTCCTTTACCATCCAAGAACTAATCTCG |
| pET28b-Spa33-C (cloning) | GCCGCGCGGCAGCCATATGAATGATAATAATGAGGCAAAAATTAATCTGTCAGAAAG | CGGAGCTCGAATTCGGATCCTTACTCCTTTACCATCCAAGAACTAATCTCG |
| pET28b-Spa33-CΔN (mutagenesis) | GAAATAATTTTGTTTAACTTTAAGAAGGAGATATACCATGAATTATGATGATATCAATGTAAAAGTGGACTTTATTC | GAATAAAGTCCACTTTTACATTGATATCATCATAATTCATGGTATATCTCCTTCTTAAAGTTAAACAAAATTATTTC |
| pETDuet-Spa33ΔRBS (cloning) | TAAGAAGGAGATATACCATGGGCAGCAGCCATCATCATCATCATCACAG | GCCGAGCTCGAATTCGGATCCTTACTCCTTTACCATCCAAGAAC |
| pETDuet-Spa33ΔRBS (mutagenesis) | GTGTAATCAAGTTATTATTGGTGATTATATTGTCAATGATAATAATGAGG | CCTCATTATTATCATTGACAATATAATCACCAATAATAACTTGATTACAC |
| pETDuet-Spa33-FL(CTD) (mutagenesis) | GTGCCGCGCGGCAGCCACATGCCGGTATTACGTAATCAAGTTTCTCTTGAC | GTCAAGAGAAACTTGATTACGTAATACCGGCATGTGGCTGCCGCGCGGCAC |
| pRSFDuet-Spa33-C (cloning) | TAATAAGGAGATATACCATGGATAATAATGAGGCAAAAATTAATCTGTCAGAAAG | GCCGAGCTCGAATTCGGATCCTTACTCCTTTACCATCCAAGAACTAATCTCG |
| pRSFDuet-Spa33-Cstrep (mutagenesis) | GAGATTAGTTCTTGGATGGTAAAGGAGTGGAGCCACCCGCAGTTCGAAAAATAAGGATCCGAATTCGAGCTCGG | CCGAGCTCGAATTCGGATCCTTATTTTTCGAACTGCGGGTGGCTCCACTCCTTTACCATCCAAGAACTAATCTC |
| pRSFDuet-Spa33-Ctrx (cloning) | ATGGTAAAGGAGTAAGGATCCGCCATGAGCGATAAAATTATTCACCTGACTG | GGCGCGCCGAGCTCGAATTCTTAGGCCAGGTTAGCGTCGAGG |
| pRSFDuet-Spa33-Ctrx (mutagenesis) | CTTGGATGGTAAAGGAGGGATCCGCCATGAGCGATAAAATTATTCAC | GTGAATAATTTTATCGCTCATGGCGGATCCCTCCTTTACCATCCAAG |
| pRSFDuet-Spa33-Cmbp (cloning) | ATGGTAAAGGAGTAAGGATCCGCCATGGAAGAAGGTAAACTGGTAATCTGGATTAACGGCG | CATTATGCGGCCGCAAGCTTTTACTGCGCGTCTTTCAGGGCTTC |
| pRSFDuet-Spa33-Cmbp (mutagenesis) | CTTGGATGGTAAAGGAGGGATCCGCCATGGAAGAAGG | CCTTCTTCCATGGCGGATCCCTCCTTTACCATCCAAG |
| pETDuet-FliM/Y  (FliY cloning) | AGAAGGAGATATACATATGACGGAAAATGAATTCCTCTCTCAGG | GTTTCTTTACCAGACTCGAGTCATTCGTTGAGAAGCTCCAG |
| pETDuet-FliM/N*  (FliN* mutagenesis) | CTTAGTATATTAGTTAAGTATAAGAAGGAGATATACATATGGTGAAGGTAGAACCGGTAGAATTCGCAGAGCTG | CAGCTCTGCGAATTCTACCGGTTCTACCTTCACCATATGTATATCTCCTTCTTATACTTAACTAATATACTAAG |
| *Fragments involved in Gibson Assembly of linear construct used to create strain GMCT113* | | |
| *sacB-kanR* fragment amplification from pIB279 | CCGGATCCTTTTTAACCCATCAC | CCCATATGTGCAAGCCTCGTCGT |
| Amplification of *spa33*-flanking upstream fragment from virulence plasmid | GTTGATACTTGATGAGTTATCACAAGAGG | ATGTGATGGGTTAAAAAGGATCCTTAGCATTCTTCTTCACTCTG |
| Amplification of *spa33-*flanking downstream fragment from virulence plasmid | GACGAGGCTTGCACATATGTAATGGTGAGTCAGAACACAC | CAGGCAAGAGGGAAAACAATG |
| *Fragments involved in Gibson Assembly of plasmids for allelic exchange* | | |
| Amplification of pKO3 backbone | TTCAATTTCATGTTCTAGTTGC | TAAACCAGCAATAGACATAAGC |
| Amplification of *spa33ΔFL* fragments from virulence plasmid | ATGTCTATTGCTGGTTTAGCCAAACAGTTGATACTTG  ATGCTAAGATAATAACATTTTGACGCTAACGAAAAAC | AAAATGTTATTATCTTAGCATTCTTCTTCACTC  ACTAGAACATGAAATTGAACAGGCAAGAGGGAAAACAATG |
| Amplification of *spa33ΔC* fragments from virulence plasmid | ATGTCTATTGCTGGTTTAGCCAAACAGTTGATACTTG  TTATTATTGGTGATTATATTGTCAATGATAATAATGAGGCAAAAATTAATCTG | CAATATAATCACCAATAATAACTTGATTACACAATAATAAATTATAAAG  ACTAGAACATGAAATTGAACAGGCAAGAGGGAAAACAATG |
| Amplification of *Δspa33*fragments from virulence plasmid | ATGTCTATTGCTGGTTTAGCCAAACAGTTGATACTTG  ATGCTAAGATAATAATGGTGAGTCAGAACACAC | CACCATTATTATCTTAGCATTCTTCTTCACTC  ACTAGAACATGAAATTGAACAGGCAAGAGGGAAAACAATG |
| Amplification of *spa33ΔFL/ΔC* fragments from virulence plasmid | ATGTCTATTGCTGGTTTAGCCAAACAGTTGATACTTG  ATGCTAAGATAATAACATTTTGACGCTAACGAAAAAC  TTATTATTGGTGATTATATTGTCAATGATAATAATGAGGCAAAAATTAATCTG | AAAATGTTATTATCTTAGCATTCTTCTTCACTC  CAATATAATCACCAATAATAACTTGATTACACAATAATAAATTATAAAG  ACTAGAACATGAAATTGAACAGGCAAGAGGGAAAACAATG |
